# New tools for immunologists: models of lymph node function from cells to tissues

**DOI:** 10.3389/fimmu.2023.1183286

**Published:** 2023-05-10

**Authors:** Tochukwu Ozulumba, Alyssa N. Montalbine, Jennifer E. Ortiz-Cárdenas, Rebecca R. Pompano

**Affiliations:** ^1^ Department of Chemistry, University of Virginia, Charlottesville, VA, United States; ^2^ Wallace H. Coulter Department of Biomedical Engineering, Georgia Institute of Technology and Emory University School of Medicine, Atlanta, GA, United States; ^3^ Department of Bioengineering, Stanford University, Stanford, CA, United States; ^4^ Department of Biomedical Engineering, University of Virginia, Charlottesville, VA, United States; ^5^ Carter Immunology Center and University of Virginia (UVA) Cancer Center, University of Virginia School of Medicine, Charlottesville, VA, United States

**Keywords:** ex vivo model, in silico model, in vitro model, organ-on-chip (OoC), 3D culture, lymphoid follicle, vaccination, human immunology

## Abstract

The lymph node is a highly structured organ that mediates the body’s adaptive immune response to antigens and other foreign particles. Central to its function is the distinct spatial assortment of lymphocytes and stromal cells, as well as chemokines that drive the signaling cascades which underpin immune responses. Investigations of lymph node biology were historically explored in vivo in animal models, using technologies that were breakthroughs in their time such as immunofluorescence with monoclonal antibodies, genetic reporters, in vivo two-photon imaging, and, more recently spatial biology techniques. However, new approaches are needed to enable tests of cell behavior and spatiotemporal dynamics under well controlled experimental perturbation, particularly for human immunity. This review presents a suite of technologies, comprising in vitro, ex vivo and in silico models, developed to study the lymph node or its components. We discuss the use of these tools to model cell behaviors in increasing order of complexity, from cell motility, to cell-cell interactions, to organ-level functions such as vaccination. Next, we identify current challenges regarding cell sourcing and culture, real time measurements of lymph node behavior in vivo and tool development for analysis and control of engineered cultures. Finally, we propose new research directions and offer our perspective on the future of this rapidly growing field. We anticipate that this review will be especially beneficial to immunologists looking to expand their toolkit for probing lymph node structure and function.

## 1 Introduction

As a secondary lymphoid organ, the lymph node (LN) serves as one of the body’s major immune checkpoints. Due to the proliferation of pathogen entry sites, humans have between 500 to 600 LNs distributed throughout the body to provide localized immune responses (1); mice, on the other hand, are reported to have between 22 to 36 LNs (2, 3). The proximal location of LNs to the cardiovascular and lymphatic systems allows for efficient antigen sorting and enables entry of immune cells from neighboring tissues (1, 4). Furthermore, the strategic localization of cells within the LN enables it to serve as a portal between innate and adaptive immunity. Indeed, a distinct characteristic of secondary lymphoid organs is the highly structured arrangement of myeloid cells, T cells and B cells, which is so evolved to produce appropriate adaptive immune responses to specific antigens (5, 6).

Historically, advances in technology have played a significant role in increasing the understanding of LN biology and its role in initiating immune responses. In the past twenty years, much has been learned from studying the LN in vivo in animal models following the advent of live two-photon microscopy. Pioneering studies in the early 2000’s and ongoing work with ever-improving imaging capabilities revealed events such as cell motility and homing, chemokine distributions, lymphocyte differentiation during trafficking, lymphocyte interaction with dendritic cells (DCs), lymphocyte migration along stromal cell networks, T cell activation following antigen recognition, and germinal center reactions (4, 7–18). More recently, the advent of “spatial biology” methods for fixed or frozen tissues, such as high-content immunofluorescence staining for proteins (19, 20) and spatially resolved analysis of gene expression (21–23), created the potential to reveal cell neighborhoods in unprecedented biological resolution.

Apart from imaging-based methods, standard approaches for in vivo studies of the LN include harvesting LNs from animals or humans for terminal analysis by flow cytometry, gene expression, or cell culture in conjunction with techniques like ELISA, PCR and Western blotting (24–26). These studies are complemented by in vitro cultures and assay methods, including experiments to track cell motility on 2D surfaces or through transwells (27–31), and to co-culture mixtures of cells, e.g. antigen-presenting cells (APCs) with T cells to study cognate antigen presentation (32, 33). Below, we briefly review what these methods have revealed about the structure and dynamism of the LN, and then present a case for the adoption of new technologies.

### 1.1 The lymph node microenvironment is intricately organized and dynamic

The LN plays a key role in host defense by mounting robust immune responses to events such as infection and vaccination, while also being critical to autoimmunity and tumor immunity. Immune functions of the LN primarily comprise maintaining naive T cell homeostasis (34) and producing adaptive immune responses, the latter mediated through pathogen surveillance, mobilizing APCs and naive lymphocytes, subduing autoreactive cells, and generating immunological memory *via* preservation of memory cells and antigens (1, 35). Each of these functions is facilitated by the confinement of cells and cognate antigens into specific niches within each LN (1). In particular, the structure of the LN is optimized to facilitate encounters between APCs and rare antigen-specific lymphocytes, thus triggering a timely adaptive immune response (1, 36).

**Figure 1:**
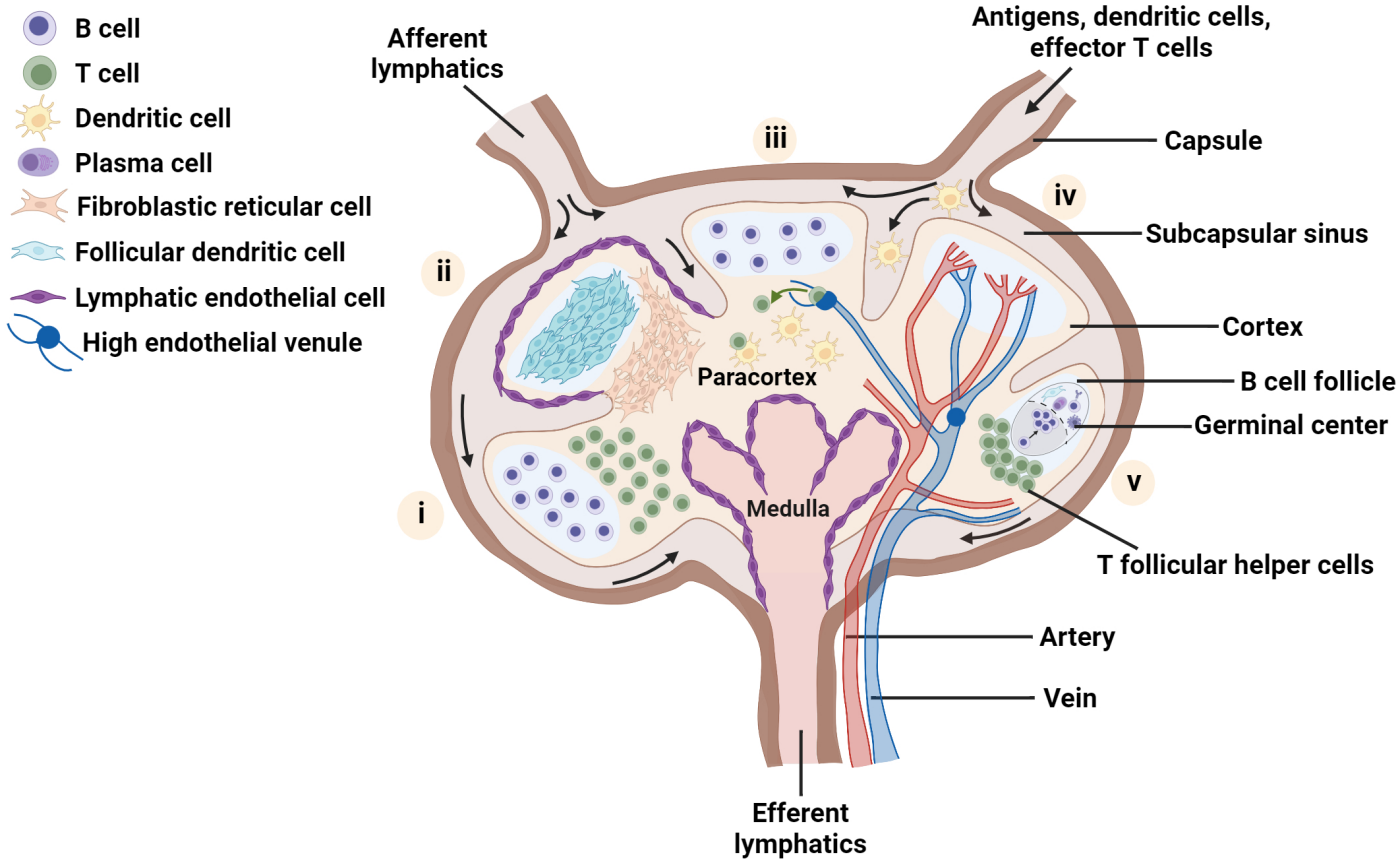
Schematic of the LN highlighting key aspects of lymph node organization and function. Each lobe shows one main feature: (i) spatial location of B and T lymphocytes, (ii) distribution of major stromal cell subsets; (iii) homing of naïve T cells into the node *via* high endothelial venules, and recognition of dendritic cells presenting cognate antigen by T cells, (iv) blood vessels within the lymph node and (v) germinal center formation. Figure created with BioRender.com.

The distinctive cellular organization that maximizes antigen-specific contact between diverse cell types is a hallmark of the LN (1, 5). Whereas most mouse LNs are 1 - 2 mm in diameter and are organized into one or two lobes, many normal human LNs are on the order of 1 cm in diameter, with a greater number of lobes. At a gross level, the LN parenchyma consists of three main regions - the medulla, paracortex and cortex – surrounded by a sinus (**Figure 1**). Located just outside the cortex, the subcapsular sinus is the first port of call for lymph fluid that enters from the afferent lymphatic vessels; the sinus is lined with lymphatic endothelial cells (LECs) and is rich in macrophages that capture incoming pathogens (37–39). The cortex, which is the outermost region of the LN parenchyma, contains B cell follicles, pockets of B cells and follicular dendritic cells (FDCs), and the interfollicular zone, which demarcates individual follicles and is host to unique APC subsets (1, 40–43). Interior to the cortex is the paracortex, or T cell zone, which contains T cells, DCs, and the fibroblastic reticular cells (FRCs) that are a critical element in the LN conduit system (1, 34, 44, 45). The medulla, located at the basal part of the LN, is characterized by a maze of lymph-draining medullary sinuses, demarcated by medullary cords, which direct lymph flow coming from the cortical sinus through the abundant efferent lymphatic vessels, thus acting as an exit route for both naive and activated lymphocytes (46–48). Cells found in the medulla include macrophages, memory T cells, antibody secreting plasma cells, and neutrophils (24, 38, 49). Blood vasculature weaves throughout the LN in a branched and dynamic fashion (50), and the LN is highly innervated as well (51, 52), with distinct organization of nerves and vessels by region.

The maintenance of spatial organization of the LN in these functionally separate regions is attributed to the presence of diverse stromal cell subtypes (5, 35, 53, 54). FRCs guide immune cell migration to and within the LN paracortex by secretion of chemical signals such as the chemokines CCL19 and CCL21, which bind to CCR7 receptors found on both B and T lymphocytes and facilitate the entry of naive lymphocytes into this region (34, 55, 56). In particular, FRCs and LECs have been shown to promote naive T cell survival in the LN *via* secretion of chemical factors such as IL-7, CCL19 and CCL21 (34). Meanwhile, FDCs and other stromal cells in and near the B cell follicles secrete chemokines such as CXCL13, which attracts B cells and T follicular helper cells and promotes their interactions in this region (57–59). Furthermore, the LN stroma has been identified as a key actor in maintaining a fine balance between the instigation of immune responses and their regulation (56, 60, 61).

### 1.2 New tools are needed to probe lymphocyte and lymph node function in controlled microenvironments

The above findings are the result of decades-long investigations into LN development, structure and function, made using tools that were each breakthroughs in their own time: light and electron microscopy (62, 63), flow cytometry and cell sorting (64, 65), monoclonal antibodies (66), transgenic and knockout animal models (67, 68), nucleic acid analysis (69), multiplexed immunoassays (70), live intravital imaging (9, 71), and so on. Today, with the growing success of immunotherapies for cancer (72) and autoimmune disease, as well as the urgent need to understand the immune response to infection by and vaccination against COVID-19 (73, 74), there is more interest than ever in understanding the role of the LN in initiating and maintaining immune responses.

Addressing this interest will require the use of modern tools to model immunity, particularly in the cases where traditional in vivo experiments fall short. For example, whereas much is known about murine immunology, it has been challenging to study the human LN in vivo, due to limited access to this organ from healthy human donors (75, 76). In addition, information on temporal dynamics has remained limited mostly to intravital imaging, due to the challenge of obtaining temporal data when the primary readouts are terminal in nature. Teasing apart the roles of individual cell types, molecules, or physical forces requires the ability to add and subtract components on demand, in defined quantities, and ideally at defined locations. Today’s wide array of analytical tools (flow cytometry, immunofluorescence, gene sequencing, -omics technologies) also demands that models of immune function should ideally be compatible with multiple readouts simultaneously.

Fortunately, some of the required tools are already available from 20 years of development in engineering, physical science, and biological science, in the form of in vitro, ex vivo, and in silico models. For our purposes, we will define in vitro models as 2D or 3D cultures of cells, such as cultures in a petri dish, biomaterial scaffold, transwell system, or microfluidic device. In contrast, ex vivo models will be defined as cultures of intact tissue that is explanted from the body, thus conserving tissue architecture. Finally, in silico models include a variety of approaches based on mathematical modeling and/or simulations, used to perform “dry lab” experiments not feasible in the wet lab. Each of these areas is still under development, and many of the newer approaches are largely untested for application to LNs or other immune organs, and still other approaches have yet to be invented.

**Figure 2:**
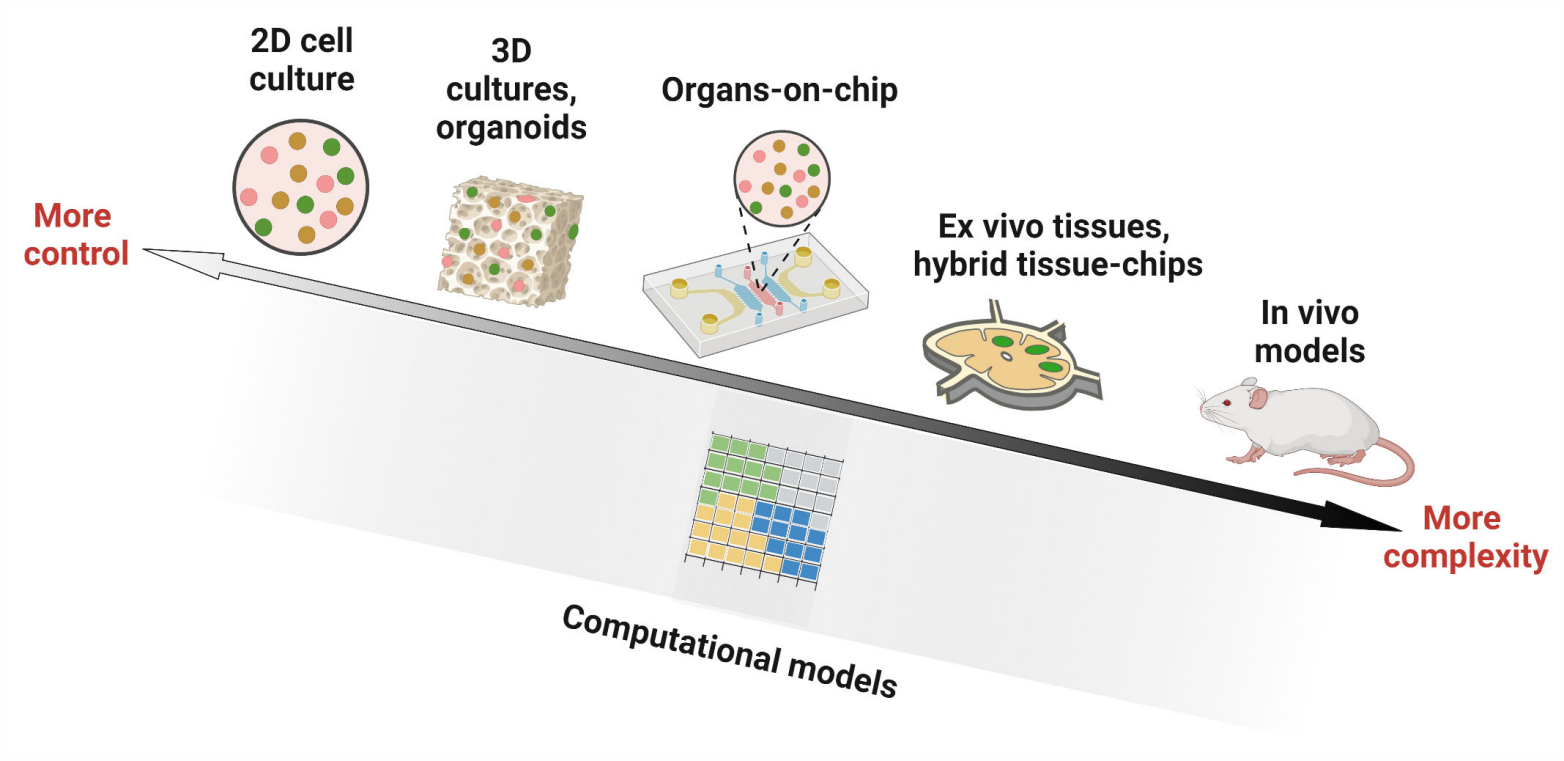
Experimental and computational tools used in studying lymph node biology. Researchers have utilized in vitro models (cell culture in 2D wells, 3D constructs such as organoids and hydrogels, and microfluidic organ-on-chip devices), ex vivo culture of tissue slices alone or in combination with microfluidic chips (hybrid tissue-chips) and in vivo models in animals, to experimentally investigate lymph node structure and function. Computational ‘dry lab’ approaches, on the other hand, employ mathematical simulations to model biological environments. They have been used to complement experimental tools and can answer questions that are challenging to interrogate by current wet lab approaches. Figure created with BioRender.com.

Any model of LN or lymphocyte function lands somewhere on an axis of biological realism versus reductionism (**Figure 2**), with associated strengths and limitations. in vivo models offer the full array of cellular and molecular players, with proper spatial organization, fluid flow, and other mechanical cues, as well as multi-organ communication. However, they can suffer from inaccessibility of test organs and more importantly, a lack of experimental control over parameters such as ligand density and selective presentation, fluid flow, or cellular or molecular composition (1). On the other hand, conventional in vitro assays provide more experimental control over which cells are present, how many, and concentration of components in the environment. This control is achieved through reductionism, in the form of assumptions and/or simplifications about cell types, extracellular matrix (ECM) components, fluid flow patterns, or dynamism of signals from outside the organ. Highly reductionist models enable ready isolation of the interactions between specific types of cells from their interactions with the rest of the organ. More complex models that allow adding or subtracting components of the organ on demand can be tested, including by adding molecular or cellular cues in specific locations and at specific times. Ex vivo models also offer intermediate complexity, with much of the experimental control afforded by in vitro cultures, along with retention of the complete tissue architecture that is found in vivo, although without connectivity to other organs. Meanwhile, in silico models range in complexity from very simple, e.g. just a few inputs and interactions, to nearly as complex as a complete tissue or multi-organ system.

While it may seem tempting to insist that a model of lymph node function is only useful if it includes every cell type (or every signaling molecule) that plays a role in vivo, such an approach is impractical and unnecessary. While adding more components to a model may potentially make it more realistic, doing so also introduces more routes for failure. Instead, each model should be designed to include only the components that are required to adequately test the hypothesis of interest. In engineering circles, this approach is referred to as a “fit for purpose” design. The converse is also true, that each well-defined biological hypothesis should be tested using a model that contains the appropriate level of complexity vs experimental control. This principle already has been applied by immunological researchers for decades when choosing between in vitro cell cultures, animal models, or human patients for each study. Now, there are simply more options to choose between (**Figure 2**), providing new capabilities.

**Figure 3:**
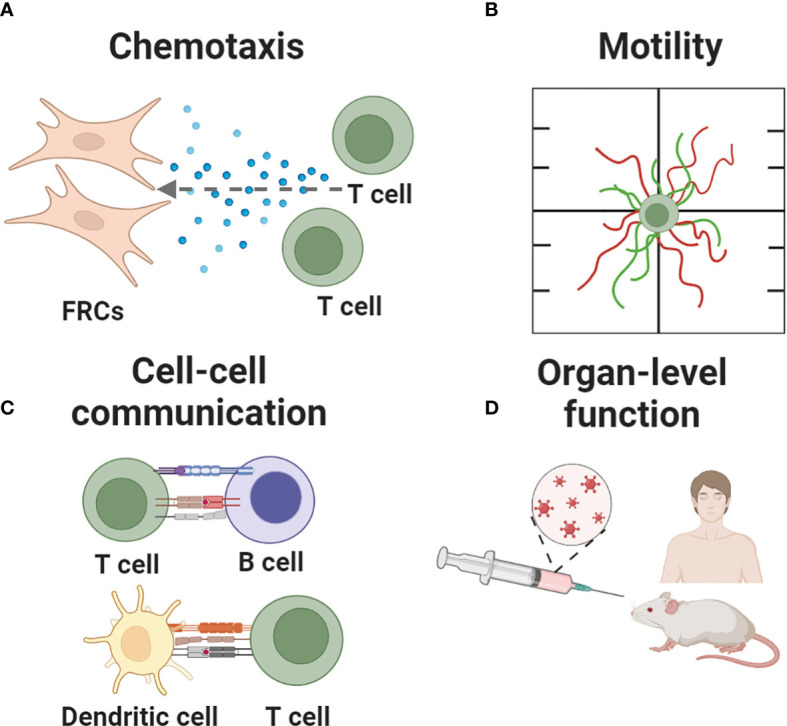
Key immune events recapitulated in current and nascent models of the lymph node. Events being modeled include **(A)** chemotaxis, **(B)** cell motility, **(C)** cell-cell communication and **(D)** organ-level functions such as fluid flow, germinal center formation, and responses to vaccination. Figure created with BioRender.com.

Here, we review the array of in vitro, ex vivo and in silico tools that are available to study and test hypotheses about specific functions of the LN. Applications of these models range from assessing the behavior of individual cells (chemotaxis, motility), to teasing apart cell-cell interactions, to predicting higher order tissue-level functions such as germinal center formation and vaccination (**Figure 3**). In contrast to recent reviews by us and others that broadly cover engineered models of immune function in health and disease (75, 77–79), this review focuses on models of the immune function in the LN specifically. To maintain a focused scope, the many excellent models of lymphatics alone, and of cancer of the LN in the absence of an immune focus, were excluded. Furthermore, with our biomedical colleagues in mind, we framed each section around the type of behavior that one may wish to model, and then present the types of tools that are well suited to tackle that challenge. For an excellent related review of LN-mimicking models organized by type of tool, we refer readers to Shou et al. (80).

## 2 Models of immune cell motility and cell-cell interactions

### 2.1 Immune cell function and cell-cell interactions

First, we will look at foundational, reductionist models of the function of individual cells or pairwise interactions that occur in the LN. By isolating individual cells or pairs of cells, their functions can be decoupled from those of neighboring cells, the ECM, and the mechanics of the microenvironment, thus narrowing down the experiment to a testable hypothesis. While there are many cell-cell interactions found in the LN, most models so far have focused on recapitulating and better understanding the processes that lead to antigen recognition. In particular, both (*i*) rapid and efficient motility of T cells, and (*ii*) establishment and capture of the T cell:APC interaction have been modeled extensively. As we will show, parameters or conditions discovered through these well-defined models can be incorporated into more sophisticated platforms later on, allowing a continuous build-up of knowledge across laboratories and fields.

#### 2.1.1 T cell and APC chemotaxis and motility in response to environmental cues

One of the signature behaviors of lymphocytes, particularly T cells, is their rapid migration within LNs and the organs they surveil (81, 82). Thus, a number of technologies have been developed to model the motility of T cells and other white blood cells, starting with simple 2D cultures (e.g. in a culture dish) (83–85). Here, we discuss newer models to study immune cell motility in the context of physiological cues, to dissect the role of factors like chemokine gradients and porosity in the LN microenvironment.

**Figure 4:**
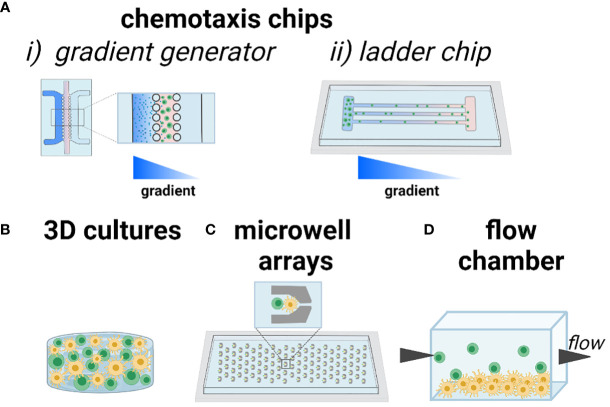
Current approaches to modeling chemotaxis and cell-cell interactions. As an illustration, T cells are shown in green and DCs shown in yellow. **(A)** Chemotaxis and cell motility are readily modeled in microfluidic channels, including (i) gradient generator (system of parallel lanes) and (ii) ladder-style chip designs to ensure organized motion for easy imaging and quantification. Gradients of chemotactic factors or nutrients are established across the chip. **(B)** 3D culture of one or more cell types, with control over the biomaterials environment. **(C)** Trapping of cells in microwells ensures that individual cells or pairs of cells can be imaged over time, in high throughput. **(D)** Seeding a 2D monolayer of cells at the bottom of a flow chamber or microfluidic channel, with cell suspension flowed by above, enables study of cell-cell interactions under physiological and pathological flow conditions. Figure created with BioRender.com.

One cell function of interest is chemotaxis, i.e. cell migration up or down a gradient of chemokine. In the LN, the recruitment of T cells to the paracortex depends on chemokines expressed by FRCs and LECs, and can be modulated by competition between chemokines, the state of the cells, or the inflammatory state of the tissue. This type of biological question is well suited for analysis using a technology sometimes referred to as a microfluidic gradient generator. Typically, cells are plated in 2D or suspended in 3D culture inside of a microchannel, with media channels on one or both sides (**Figure 4A**). By continually adding chemokine-loaded media on one side, and control media on the other, a stable gradient is established across the culture, and the chemotactic response can be imaged in real time or as an endpoint assay. This technology was first described in the mid 2000’s (86) and is now well established and user friendly, with commercially available devices (87) ready for loading by simple pipetting. As an example, many T cells and DCs are recruited to the deep paracortex by CCL19 and CCL21, which share a common receptor, CCR7. While CCL21 is present at >300x greater concentration than CCL19 in the LN (88), their relative effects on T cell motility are difficult to dissect in vivo. To investigate their interactions, a microfluidic gradient generator with a Y-channel geometry was loaded with human T cells pre-activated with anti-CD3/CD28 antibodies (89). While activated T cells exhibited similar mean velocities in the presence of uniform CCL21 or CCL19 gradients at physiologically relevant chemokine concentrations, directional migration occurred towards a CCL21 gradient, but away from a CCL19 gradient in the presence of a homogeneous CCL21 concentration field. From these data, the authors suggested a combinatorial guidance mechanism for T cell migration within the LN; while both CCL21 and CCL19 have similar binding affinity to CCR7 receptors, CCL19 strongly desensitizes and internalizes CCR7.

A related approach to studying chemotaxis is to subdivide the cell culture area into restricted lanes or channels, so that cells must migrate in an organized manner that facilitates imaging and quantification (**Figure 4A**). This type of device is sometimes referred to as a microfluidic ladder chip, and was described early on for applications ranging from neuronal axonal growth to neutrophil and lymphocyte motility (90–92). The ladder is usually coupled with the introduction of a chemotactic gradient to induce directional motility. For example, this type of system was used for studying antigen transport and presentation by DCs in structured co-culture with T cells. Two parallel compartments containing human-derived DCs and primary blood-derived T cells respectively, were separated by a series of small transversal channels (93). In response to an applied CCL19 gradient, mature DCs chemotaxed towards the T cell compartment whereas immature DCs did not. Engagement of mature DCs with T cells induced T cell activation, which was confirmed *via* intracellular calcium signaling.

Biomaterials-based 3D cultures have also been developed to study T cell motility in response to environmental cues (**Figure 4B**). For example, a 3D culture model of human T cells and human-derived matured DCs in agar gel showed that T cells exhibited a random walk without directional bias in response to a CCL19 gradient, whereas DCs exhibited chemotaxis towards the same gradient, thus demonstrating clear differences in responses by T and DCs to CCL19 (94). Beyond applying chemokine gradients, biomaterials can be used to control molecular and physical properties such as integrin ligation, stiffness, and porosity (95, 96). The capabilities of such materials go far beyond those of common materials such as agar and Matrigel, which are biological products with poorly defined composition. For example, to test the conditions under which primary T cells migrate efficiently, a custom ex vivo scaffold was developed with a tunable structure and composition (97). Specifically, an inert polyethylene glycol (PEG)-based hydrogel was conjugated with heparin (for cell and chemokine adhesion through electrostatic interactions) and cast over sacrificial beads of controlled diameter, a process called colloidal templating that once dissolved, generates a macroporous gel. The pores were subsequently filled with a fibrillar collagen matrix, through which lymphocytes could crawl. The scaffolds were loaded with murine CD4 T cells and mature DCs. The diameters of the pores proved to be particularly influential in both the velocity and maximum displacement achieved by T cells, with larger pores (80 µm) resulting in the highest amount of pore-pore trafficking by T cells. These data have been used to inform subsequent scaffold design to ensure T cell motility (98). Interestingly, DCs were observed to have heterogeneous levels of migration within the scaffolds, but unlike T cells, the migratory DC population could get through pores as small as 10 µm. Custom materials like this one (99) are generally not yet commercially available, but this is changing as the demand for them grows, and likely more will become available in the next few years.

#### 2.1.2 Interactions between T cells and antigen-presenting cells

Once a naïve T cell encounters an activated APC that displays its cognate antigen, the engagement between the T cell receptor (TCR) and peptide-major histocompatibility complex sets off cellular responses that may eventually lead to adaptive immunity. Activated APCs may also modulate T cell activity *via* a “bystander” mechanism, e.g. by secreting cytokines that act in a paracrine manner on nearby cells. In the LN, notable APCs include DCs and B cells. A number of technologies have been developed to study this interaction, for example to decouple the role of physical contact from secreted factors.

One of the earliest engineered platforms developed to observe the interaction between specific cells in vitro relies on microwell arrays (**Figure 4C**). By capturing cells sequentially, either by microfluidics or gravity, it is possible to assemble two or more cells in each well (on average), with defined cell ratios. Standard microfabrication approaches easily generate arrays with hundreds or thousands of cells, thus allowing imaging of the behavior of individual cell pairings over time (100). The exact number of cells captured per well in each cycle is usually controlled by a Poisson distribution: to ensure no more than one T cell per well, for instance, a low density of T cells is applied, yielding 0 or 1 cell per well in the majority of wells (101, 102). As an example, this strategy was used to test the effect of paracrine conditioning prior to physical contact between human T cells and in vitro matured DCs (mDCs) (103). After passively trapping the cells in two separate devices, the devices were coupled in a daisy-chain manner, so that the mDCs’ effluent flowed downstream to an array of naïve T cells. By monitoring the calcium response in real time after addition of mDCs to the T cell array, it was shown that calcium transients were reduced in cells preconditioned by DC effluent. Microwells have also been used to test the interaction between CD8+ T cells and pre-activated B cells (104). These early studies demonstrated the power of an array of cells confined in space, enabling single cell and single-pair longitudinal analysis, and revealing heterogeneity in the response of sub-populations of T cells that could be averaged out in bulk measurements (100). Microwell array chips are often easy to use, sometimes requiring only simple pipetting to load, are commercially available (105–107), and are usually designed for compatibility with standard microscopes.

In vivo, T cell-APC interactions occur under varied interstitial flow rates (108). To test the effect of flow and shear stress on cell-cell interactions, microfluidic flow chambers provide straightforward control (**Figure 4D**). For example, by culturing a monolayer of antigen-pulsed murine DCs and introducing antigen-specific CD4+ T cells under continuous flow, the threshold shear for antigen-mediated engagement was tested (109). At the physiological shear stress of 0.01 Dyn/cm^2^, T cell–APC interactions matched the mean duration (12.8 min) and intermittent crawling with neighboring DCs seen in vivo, demonstrating the influence of shear stress in T cell priming and activation.

To continue investigating the interconnected functions of T cells and APCs in the paracortical region, numerous groups have complemented the wet lab with computational models. Due to the dense, spatially complex, and highly motile microenvironment in a LN, it can be difficult to elucidate the rate-limiting steps and migration dynamics of T cells and APCs through experiments alone. Computational models offer an avenue to ask biological questions about the LN that are impractical or too resource-consuming in vivo or in vitro, e.g. by varying cell activation states, migratory parameters (velocity, chemotactic response etc), while maintaining or varying biomimetic fluid flow rates. These models are sometimes developed to answer specific questions. For example, using animation software and assumptions drawn from experimental images, in 2004 Catron et al. (110) produced a video that simulated a lymph node slice and illustrated naïve CD4+ T cell engagement to antigen. The video tracked the random motion of naïve CD4+ T cells, B cells and DCs in the absence of antigen, and tested how lymphocyte motility changed after encountering soluble antigen. Vroomans et al. (111) developed a tissue level model to test the role of chemotaxis and antigen specificity in the efficiency of DC scanning for cognate T cells. By comparing scanning efficiency with and without chemotaxis, the model predicted that highly localized chemotaxis of T cells towards DCs improved scanning efficiency, in part by recruiting more T cells to each DC, while still retaining the characteristic features of a random walk when observed at the tissue scale. Other models are built to capture a larger number of aspects of the system, thus facilitating broad use for a variety of hypotheses. For example, Bogle and Dunbar developed an agent-based model to study T cell “random walk” motion, including cognate interactions with APCs, chemotactic gradients, T cell ingress/egress in the paracortical space, and vascular remodeling due to inflammation (112–114). An agent-based model is one in which individual cells act as autonomous, decision-making entities in a defined grid-like microenvironment; it is well suited to modeling cells in tissue. Their approach employed a 3D lattice structure for T cells to be seeded onto, and was designed to parallel experimental intravital microscopy data from the LN paracortex while omitting FRCs for simplicity (112). An agent-based model by Azarov et al. (115), further explored T cell migration behavior and interactions with DCs, with a focus on time scales and spatial limitations.

A complete tutorial on computational models of LN function is beyond the scope of this review. We point readers to Mirsky et al. (116), which details a variety of types of models of the LN during acute and chronic inflammation, with an emphasis on the conventions for inclusion and exclusion of select cell populations, time dependent flux of cell densities, dimensionality (e.g. 2D vs 3D), steady state assumptions, and other simplifications. A more recent review by Novkovic et al. (117) discusses computational investigations into LN organization and function with a particular focus on the structural contribution of stromal cells and their roles in chemotaxis and lymphocyte migration. Just as in experimental model systems, it is important to clearly define the assumptions and limitations underlying each computational model, to allow informed use of the predictions in translation to physical systems. Implementation of computational models currently requires varying levels of coding, from minimal with beginner-friendly software such as Netlogo, to more advanced professional systems. Using such tools, computational experiments have potential to answer questions that are cumbersome to test in vivo, and to make predictions that can then be tested in animal models.

### 2.2 Interactions between T cells and the lymph node stroma

The stroma is critical for preserving the structural integrity and function of secondary lymphoid organs (5, 118). LN stromal cells play key roles in lymphocyte survival and migration, lymph transport, nutrient and antigen supply, immunological monitoring and mediation of adaptive immune responses (35, 53). Inclusion of stromal cells in experimental models is likely to be particularly essential for replicating organ-level function and investigating immune responses to events such as antigenic challenge and vaccination. The majority of current knowledge on the LN stroma has been uncovered through in vivo immunology experiments (4, 25, 34, 45, 119–121), including elegant studies into how stromal cells and their networks define the structure of the (mostly murine) LN microenvironment. Presently, there are limited instances of in vitro or ex vivo models incorporating stromal cells in the literature – more research effort is required in this regard.

To investigate the role of the human lymphoid stroma microenvironment in T cell activation, recently in vivo animal models were complemented by in vitro culture of human T lymphocytes on monolayers of human FRCs and ex vivo culture of human tonsil slices (92). FRCs inhibited proliferation and controlled differentiation of newly activated T cells independent of feedback signaling from the lymphocytes. This effect was mediated by four molecules: cyclooxygenase 1, transforming growth factor beta, adenosine 2A receptor and indoleamine-2,3-dioxygenase. It is noteworthy that these results may have been difficult to obtain in vivo, where interactions between specific cell populations (T cells and FRCs) are challenging to isolate. The use of intact human tissue slices in the study was unique as most studies often use dissociated human cells or murine cells/tissue. In addition, this approach enabled validation of in vitro experiments *via* in situ T cell activation studies.

Although stromal cell organization plays a critical role in LN function, it remains unclear how spatiotemporal variations in stromal cell density, network geometry, ECM deposition, and expansion of the LN during an immune response may direct the migration and proliferation of lymphocytes and APCs. These questions are challenging to address directly in vivo. Thus, efforts have been made to replicate certain attributes of the LN stroma and key events therein using in vitro 3D models. Early work by Katakai et al. carefully characterized in vitro co-cultures of LN stromal cell lines and T cells in 2D, culminating in an early transition to 3D culture by seeding LN stromal cell lines in a nylon mesh (121). More recently, by encapsulating human bone marrow stromal cells in fibrin and collagen hydrogels, Kim et al. (122) developed a 3D stromal model for co-culture with T cells for use as a tool to study cell-cell/stroma interactions during immune events. Of note, network formation of stromal cells in vitro often induces contraction of soft 3D cultures; here, degradable fibrin was included to counter the contraction, and a carefully titrated protease inhibitor was included to control the rate of degradation and keep the gel stable. Following CD4 T cell addition to the model, T cell adhesion and migration along stromal networks was detected and comparable to T cell zones in vivo. This type of 3D culture model is poised for use to test hypotheses in the future, making use of the spontaneous formation of stromal networks. A key advantage of using in vitro models to answer biological questions lies in their tunability and potential to recapitulate cell-cell/ECM interactions and complex events.

While spontaneous network formation is a convenient feature of standard 3D culture models, it can be challenging to directly control parameters such as the connectivity of the network in such models. Here, computational models of stromal networks can fill a major experimental gap. For example, Novkovic et al. (123) used “graph theory” to model the interconnectedness of structural networks and their impact on T cell and DC migration. Application of this model concluded that the FRC network in vivo exhibited “small world network” characteristics with a high degree of FRC clustering and loss of network function when 50% of FRCs were removed.

Sometimes, computational models can highlight a gap in the understanding of the biological system. In a model focused specifically on T cell migration, Beauchemin et al. (124) and Beltman et al. (125) used a three-dimensional Cellular Potts Model to evaluate T cell motility in relation to the FRC network, comparative to in vivo two-photon microscopy data. A Cellular Potts Model is a lattice-based method that allows for the study of diffusion, migration, and intercellular interactions. Results suggested intentional movements over shorter periods, overall random movement over long periods, and the possibility of “T cell streams” in which cells were influenced to travel in the same direction in discrete periods, contradicting the standard notion that T cells must adhere to the FRC network (4). This is a prime example of both the pros and cons of computational models. We find ourselves in one of two scenarios: either (i) computational data offers us a new explanation for physical phenomena or (ii) we are offered an explanation that does not match reality due to simplifications made by the model’s designers. While we cannot elucidate which statement is correct on its own, additional investigations will be necessary to test the validity of the simulation’s predictions in a physical setting. Thus, computation can offer many opportunities, including the lessons learned while seeking to externally validate their predictions, and are highly impactful in their ability to bolster innovative problem-solving and high-throughput processing of experimental directions and immune therapeutics design.

## 3 Models of specific organ-level functions

### 3.1 Modeling fluid flow and diffusion through the lymph node

In vivo, the LN is continually bathed in flow of lymphatic fluid, which arrives from the afferent lymphatic vessels, passes through the sinuses and in part through the parenchyma, and finally out through the efferent lymphatic vessels. For many years, the potential role of fluid flow was largely omitted from immunological dogmas, but pioneering work by Melody Swartz and others over the past 20 years revealed the significant immunological implications of flow both in the lymphatics and in interstitial tissues in the LN and elsewhere (126, 127).

**Figure 5:**
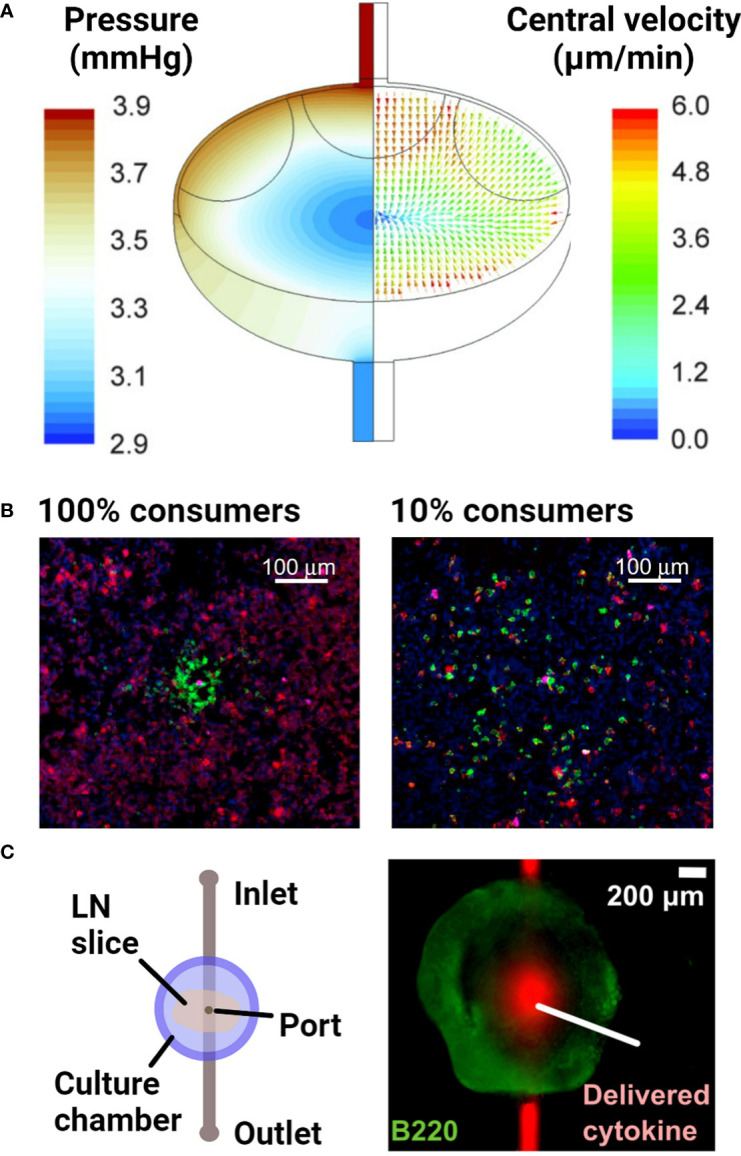
Examples of models of fluid flow and diffusion in the lymph node. **(A)** A computational fluid dynamics model, based on confocal microscopy images of the lymph node, predicted the advective fluid flow paths and velocities through the lymph node. The majority of the fluid flow passed around the edges of the node, in the sinus regions. Reproduced with permission from (132). Copyright 2015 Mary Ann Liebert, Inc. **(B)** Densely packed 3D cultures of reporter T cells predicted the distance over which secreted cytokines diffuse and elicit responses. Varying the proportion of IL-2 consuming T cells (100% vs 10% consumers) around IL-2 producing T cells indicated that IL-2 secretion was non-directional in this setup. Cells were stained with DAPI (blue) to identify nuclei, anti-IL-2Ra (green) to identify IL-2 consuming T cells and anti-pSTAT5 (red) to detect T cell response to IL-2. With greater consumption (100% consumers), IL-2 secretion remained more localized and had a smaller magnitude of signaling. Reproduced with permission from (135). Copyright 2017 Elsevier. **(C)** Schematic of a microfluidic chip developed to track diffusion of cytokines after delivery into live lymph node tissue slices through a port of entry. Reproduced with permission from (136). Copyright 2018 Elsevier.

Some of the first questions that may be asked about flow through the LN are what path it takes, at what velocities, and how these change over time or upon inflammatory or other stimulation of the LN. Flow rates through lymphatic vessels have been measured directly in vivo, e.g. by cannulation in larger animal models (128–130). However, interstitial flow velocities and paths through the LN parenchyma have been much more difficult to quantify (see O’Mallia et al. (127) for an excellent recent review). So far, the best quantitative estimates have come from a combination of in vivo imaging with computational models, which enabled fits of experimentally measured incoming flow velocities or pressures to image-based geometries or an idealized version of LN organization. The first two such models of the whole LN appeared in late 2015 (131, 132), and continue to be further developed since then (133, 134). Both models predict that the fastest lymph flow occurs in either the subcapsular or transverse sinuses, and that the majority (90% in (132)) of the incoming lymph fluid likely passes through the sinus rather than through the densely packed parenchyma (**Figure 5A**). The exact numerical values of predicted velocities through the interstitium should be interpreted cautiously, as they strongly depend on a parameter called fluid permeability (Darcy permeability) or hydraulic conductivity, which describes the ability of fluid to flow through porous matrices like tissue. Permeability has not been well characterized in the LN, and likely varies over time and across the tissue, as a function of the local cell density and ECM composition (137). Nevertheless, using reasonable approximations and/or fits for this parameter, the models provide reasonable predictions of flow velocities on the scale of mm/min in the low-resistance sinuses, and µm/min in the packed parenchyma. So far, these models have been generated based on images and experimental data taken from healthy LNs; they have the potential to be extended to inflammatory and disease conditions if sufficiently resolved imaging and measurements of afferent or efferent flow become available.

Dramatic changes in size, cell content, stromal organization, and fluid flow all occur during inflammatory responses (120), and there is good reason to expect that changes in flow rates in the LN will have a substantial impact on the biology of this organ. However, so far, direct experimental tests of this hypothesis at the tissue level are few. Interestingly, in ex vivo LN slice cultures, perfusion of the tissue improved lymphocyte motility during live two-photon imaging, though it was not clear whether the effect was rooted in mechanical stimulation or oxygen delivery (138, 139). Using a more reductionist approach, a number of well-defined in vitro and in vivo experimental models have been developed to test the effect of flow on specific cell types or subunits of the LN or lymphatic system, reviewed in (127). Such tests showed that LECs and FRCs in the LN stroma are sensitive to flow and shear stress, upregulating chemokines such as CCL21 and CCL19, cellular adhesion markers, and cytokine expression under physiologic flow conditions (140–142). T cells also respond to shear flow with increased adhesion to endothelial walls and improved motility (143, 144).

One approach to investigating how fluid flow influences FRC organization and function is to use transwell-based 3D cultures to recapitulate the LN stroma. For example, Tomei et al. (142) used murine FRC clones that were grown in collagen/Matrigel-embedded polyurethane sponges, and applied interstitial flow using a simple gravity-based system. Application of slow interstitial flow improved FRC organization and alignment with the ECM in addition to increased CCL19 and CCL21 secretion. Furthermore, low shear stresses directly stimulated CCL21 secretion. These in vitro findings were validated in the same paper by in vivo studies in mice, which showed that CCL21 transcription was significantly reduced when the afferent lymphatics leading to the LN were surgically cut, although it was not possible to distinguish the impact of lost fluid flow from any loss of cellular trafficking or an acute response to the wound. This was one of the earliest papers to consider the effect of fluid flow on the functionality of LN stromal cells and has inspired many groups to continue in this area. So far, no additional reports have replicated the finding that in vitro fluid flow induced CCL21 or CCL19 expression or secretion by cultured LN stromal cells, perhaps because of differences in cell sourcing. Nevertheless, transwell-based 3D cultures are an excellent means to apply gravity-driven or pressure-driven flow to cells of interest; and are easily accessible to any biomedical research lab (145).

Another means of testing the effect of fluid flow on lymphatic and lymphoid cultures is to make use of microfluidic devices. These devices can be coupled with syringe pumps or peristaltic pumps for well-controlled flow rates, or with gravity-driven or other passive flow control systems for a simpler approach (146, 147). There has also been progress in generating parallelized microfluidic devices, e.g. by integrating them with multi-well plate formats, to enable high-throughput drug screening (148). Although models of lymphatics have been developed (149–151), microfluidic flow has been applied only recently to tissue-level models of the LN. In one recent study, Birmingham et al. (152) developed a clever microfluidic platform that recapitulated the varied fluid flow rates and geometries of the sinus encountered by metastatic cancer cells upon entering the LN. By functionalizing the channel with different adhesion receptors, they observed that increases in the flow and shear increased levels of E-selectin-mediated metastatic and monocytic cell adhesion. Thus, this model highlighted the potential biological impact of small changes in sinus height and lymphatic vessel diameter, structural changes that are known and expected in the context of human disease, on cell behavior in the lymph node.

Aside from investigating the impact of fluid flow on lymphocyte function, the key role of cytokine and chemokine gradients in driving lymphocyte behavior has resulted in efforts to understand the formation and maintenance of gradients and local activity in the LN. Answering this question requires replicating the transport of secreted proteins by diffusion alone or diffusion with flow, in combination with their binding to cell surface receptors or the ECM; the latter sometimes leads to internalization or deactivation. Experimentally, one strategy has been to use densely packed, matrix-free lymphocyte cultures in the bottom of a small well or a 3D stack of cells. This approach was used to demonstrate that cytokines are active within just a few hundred microns from the cell that secreted them (**Figure 5B**), and the results were verified in vivo (135). For more physiological organization and retention of the extracellular matrix, LN slices have been coupled with a simple microfluidic chip (**Figure 5C**) to quantify diffusion coefficients of various cytokines in specific regions of the tissue (136).

Meanwhile, computational approaches that combine diffusion, secretion, and binding are a staple and are sometimes referred to as “reaction-diffusion models”. This computational approach has been implemented in LNs by considering the various biological compartments of the organ, resulting in predictions of dynamic cytokine distribution that would be difficult to achieve from in vivo measurements (153). More recently, chemokine diffusion was integrated into one of the fluid flow models of the LN discussed above, to predict the impact of disrupted lymphatic flow on the development of chemokine gradients (154). Whereas so far, all experimental in vitro models of fluid flow in the LN have been simplified to an assumption of constant flow rates, computational models have begun to incorporate the pulsatile flows observed in vivo. The findings of computational models inform not only the fundamental biology of the LN, but also support the development and validation of novel in vitro experimental systems (e.g. organ-on-chip models) by providing benchmarks for flow rates, shear forces, and molecular transfer that is otherwise hard to obtain.

### 3.2 Models of B cell follicles and germinal centers

In the LN, humoral immune responses to antigens are driven by B cells located in follicles (43). Upon antigen presentation by APCs or direct antigen encounter, naive B cells differentiate into antibody-secreting plasma cells, memory B cells and long-lived plasma cells through the germinal center reaction, a three-step process involving clonal expansion, somatic hypermutation and class-switch recombination (43, 155, 156). Together, these cell populations mount systemic antibody-driven responses to infection by reducing pathogen spread and provide lasting protection following vaccination (19). Recapitulating B cell interactions can be useful for probing how adaptive immune responses to infection and vaccines are mediated in the LN.

To understand how cell-cell interactions drive secondary lymphoid organ development, Suematsu and Watanabe (157) implanted lymphoid tissue-mimicking collagen organoids containing DCs, T and B cells into the renal subcapsular space of mice. Over time, the implanted organoids recapitulated characteristic secondary lymphoid organ hallmarks including distinctive T and B cell clusters, FDC networks and germinal center formation. In addition, antigen-specific IgG1 antibody production was detected after organoid re-transplantation to naïve or severe combined immunodeficiency mice, which was indicative of B cell class switching and differentiation. Later studies showed that the artificial organs were immunologically active, eliciting strong antigen-specific immune responses upon a second encounter even without stromal cells present (158, 159).

Considering the ethical barriers, costs and complexities of conducting in vivo implantation studies, efforts have also employed in vitro models for interrogating how the lymphoid microenvironment drives germinal center reactions and the consequent antibody production. To generate functional ‘synthetic immune tissues’ that could replicate crucial germinal center reactions, Purwada and colleagues developed in vitro organoids. B cells were co-cultured with stromal cells that were engineered to express ligands normally found on T follicular helper cells and FDCs, in a hydrogel matrix of nanoparticle-modified gelatin (160) or modified PEG (161). This strategy enabled tests of how integrin ligand type and density impacted germinal center-like outcomes without requiring in vivo models. Further work demonstrated that the PEGMAL organoid components could be tuned to regulate germinal center activation and achieve selective enrichment of antigen-specific B cells (162). In another study, Goyal et al. (163) developed a microfluidic flow chamber to mimic the clusters of B cells seen in lymphoid follicles. The two-chamber microchip contained primary human T and B cells cultured in Matrigel/collagen hydrogel in the bottom chamber. Media perfused through the top chamber induced self-assembly of T and B cells into 3D aggregates and flow-directed organization of collagen fibrils. These cell aggregates behaved similarly to off-chip high-density cultures, with the potential in the future to use the chip to vary parameters such as flow rate or cell organization.

Like the paracortex, the complexity of B cell germinal cell differentiation has also been explored through computational models. Martínez et al. (164) established a kinetic model of B cell germinal centers, based on ordinary differential equations, accounting for the gene expression, gene regulation, and direction of terminal differentiation of B cells, with particular focus on B cell receptors and CD40 signaling. This model was extended to lymphomagenesis, especially in B-lymphocyte-induced maturation protein 1 (BLIMP1) inactivation repressing apoptosis and facilitating cancerous cell proliferation. This model was further adapted and modified by Thomas et al. (165) to consider stochastic extracellular events, with particular focus on the role of interferon regulatory factor 4 in relation to memory B cell differentiation, but with cells characterized by probability in relation to their migration in or out of the germinal center. This is especially important for predicting the progression of diffuse large B cell lymphomas and how targeted gene therapy of BLIMP1 or other such genes can impact lymphomagenesis or work to repress it, paving the way for a faster development of clinical anti-lymphoma treatments. The investigation of germinal center B cell differentiation and class switching in relation to defined signaling cascades, such as BLIMP1, may be especially useful to those using in vitro lymphomagenesis models. Specifically, these models could quickly determine the level of promise of certain targets *via* high-throughput computation, resulting in conserved time and resources to prioritize the best candidates and potentially revolutionize the development of anti-lymphoma vaccines and therapeutics.

## 4 Applied models of vaccination and immunogenicity

Apart from probing LN structure and function, models of the LN have also been developed to test specific biomedical applications, particularly for vaccine development, adjuvant testing, and predicting the immunogenicity of antibody therapeutics. This area is still in early stages, as no system yet has been demonstrated to predict immunogenicity of novel antigens, but exciting progress has been made towards this goal. Below we discuss three complementary approaches, which differ in the level of complexity and control over their biological inputs and that of the microenvironment (**Table 1**).

**Table 1:** Properties of three approaches to model tissue-level functions in the lymph node, compared to in vivo analysis.

	In vivo in animals, humans	Live ex vivo tissues	Cell suspensions, organoids	Engineered systems
**Fluid flow**	✔			✔
**Lymphocyte (re-)circulation**	✔			✔
**Primary spatial organization of cells, proteins, ECM**	✔	✔		Determined by cell input^†^
**Diversity of cell populations retained**	✔	✔		Determined by cell input^†^
**Drug delivery at known doses, locations, times**	No (subject to pharmaco-kinetics)	✔	✔	✔
**Repeated analyses in same sample**	Limited; many assays are terminal	✔	✔	✔
**Analysis of protein secretion in situ**		✔	✔	✔
**Compatible with widefield imaging**		✔	✔	Depends on platform
**Precise control over cell and protein content**			✔	Determined by cell input^†^
**Low variation between replicates for drug screening**			✔	Determined by cell input^†^

^†^Determined by whether tissue explants, cell suspensions, or organoids are used, and how these were prepared.

### 4.1 Models using intact primary tissues

**Tonsil slices** have long been used to study human immunity in the context of immune events such as infection, vaccination and antigen challenge (166–170). While tonsils have a greater frequency of germinal centers and increased T cell activation compared to other LNs due to constant exposure to the microbiota and antigens in the mouth (171), they are one of very few sources of human LNs readily available as fresh surgical discard tissue. An advantage of working in slices compared to well-defined reductionist models is the retention of all cell types that were present in vivo at the time of collection, as well as their spatial organization (172). This feature imparts substantial heterogeneity between slices in terms of both cell composition and spatial organization, as each slice comprises only a fraction of the larger, highly structured organ (173). Pioneering work by Margolis et al. showed that human tonsil and other LN slices made a powerful model of viral infection, including e.g HIV replication and virus-specific class-switched IgG production (174–176). Use of tonsils to model infection continues today (166–170). Tonsil slices have also been used to reveal fundamental LN biology, such as in work by the Fletcher lab, who used tonsil slices to confirm a regulatory role for the LN stroma in T cell activation (118).

In some cases, animal models provide access to tools not available with human tissue, including TCR transgenic models of known antigen specificity, reporters, and other genetic tools. Murine LN slices have been used extensively to study T cell motility (28, 177–180), and more recently to study higher order functions. Belanger et al. (181) characterized this model system in detail, including viability, absence of slicing-induced inflammatory gene expression, T cell response to crosslinking of the TCRs, and response to toll-like receptor ligation. Importantly, LN slices collected from mice previously vaccinated in vivo responded to ex vivo antigen challenge with expected T cell cytokine secretion and activation markers. Interestingly, skin-draining LN from vaccinated mice did not adhere as well to the embedding matrix (agarose), and this change could be mitigated by a brief treatment with mild detergent, suggesting a change in the lipid composition of the exterior LN capsule (182). As skin-draining LNs in mice are embedded in fat pads, this change may reflect a broader change in the adipose tissue environment; this observation remains to be explored. Murine LN slices have been combined with microfluidic environmental control for local drug delivery to T cell zones and B cell follicles (183), to quantify molecular diffusion in the LN (136), and to form a tumor-LN co-culture model (184). Ramirez et al. (185) combined ex vivo LN slice cultures with PEG nanoparticle tracking to characterize interstitial tissue properties such as pore size and viscosity. Tracking studies indicated that LN pore sizes were highly variable, while microrheology showed that the LN extracellular tissue was viscoelastic and assumed hydrogel-like properties. In addition to microscopy-based readouts, flow cytometry, and ELISA, ex vivo LN slices are also compatible with *in situ* analyses that would be challenging to conduct in vivo, such as electrochemical measurement of neurotransmitters and hormones (186). A recent report of murine spleen slices with excellent retention of spatial architecture opens the possibility for work in that organ as well (187). Reports of intact explant culture of LN or spleen from large animals remain rare (188), but are of significant interest for models of immunity in veterinary patients and livestock (189).

Recently, ex vivo culture of intact tissue blocks was compared to organoid cultures in a systematic series of tests of germinal center formation, response to antigen, and response to anti-inflammatory therapies, starting with human adenoid tissue (168, 190). In that study, although the tissue blocks better retained the germinal center structure, they were not as responsive to manipulation of CD40L and IL-4 signaling pathways as the organoids, and were comparatively less viable after just 3 days. However, it is possible that the large size of the blocks (∼1 mm in the smallest dimension) may contribute to these challenges. The expected penetration distance of oxygen in tissue is typically limited to 200-400 μm, depending on the cell density and metabolic activity of the tissue (191). Head to head comparison of culture systems remains rare, and further study to determine how smaller tissue slices may compare to organoid-style systems for various functions would be valuable.

### 4.2 Models of tissue-level activity using suspensions or organoids

Tissue-derived models rely on the availability of fresh human or animal tissue, both of which can be limited. A complementary approach is to develop bottom-up models of LN function by combining specific cells in vitro. Most of these models begin with primary leukocytes, frequently collected from donations of whole blood or from the leukopaks that are generated as leftovers from platelet donation (192). White blood cells have been combined with cell lines to represent endothelium or stroma as needed. Other models begin with cells obtained from secondary lymphoid organs after surgical resection. For a discussion of considerations in cell sourcing that are unique to models of immunity, we refer the reader to Hammel et al. (79). Despite intense interest for at least 20 years from funding agencies such as DARPA, NIH, and FDA, models of vaccination in the LN have so far been limited to just a few systems. Below we review developments so far in this exciting area.

As mentioned above, one approach to modeling lymphoid function is to isolate cells directly from human blood or from surgically resected tissues and generate homogeneous high-density cultures or organoids. These may be generated simply by pipetting a high density of cells into a well plate, sometimes using V-bottom or U-bottom plates to further encourage high density. For example, T follicular helper cells derived from naïve T cells in the blood may be co-cultured with isolated memory B cells, for a system that nicely replicates plasmablast formation and IgG production (193). Blood-derived cells are readily obtained, but they may differ in phenotype from tissue-resident cells. Therefore, cultures and organoids generated from tonsils, adenoids, and other LNs have been explored as well. For example, culture of adenoid-derived cells in a well plate successfully generated physiological responses such as proliferation, germinal center B cell formation, and antigen-specific T cell cytokine secretion in response to signals such as cytokines, small molecule drugs, recall antigens from prior vaccinations, and immunotherapies (168). Similarly, culture of LN cell suspensions from *Leishmania major*-infected mice proved an effective method to support proliferation of the parasite and to rapidly screen antiparasitic drugs (194).

Recently, tonsil organoid cultures were generated by allowing tonsil-derived cells to reaggregate atop a transwell membrane over several days; these were used to study response to vaccines (195). The organoids included regions of B cells that were reminiscent of the light and dark zones of a germinal center. The cultures responded to stimulation with recall antigens such as influenza and the measles, mumps and rubella (MMR) vaccine with IgG secretion, B cell maturation, and T cell activation. Excitingly, the organoids were also able to respond to antigens that the tissue donors were likely naive to, including rabies vaccine and COVID-19 vaccine candidates, though mostly with IgM and not IgG secretion. Overall, this study demonstrated that immune organoid cultures may offer the potential to scrutinize certain vaccine candidates.

Comparing tissue slices versus high-density cultures and organoids, the former has the benefit of retaining the original spatial organization and stromal networks of the organ, which is a key strength for mechanistic studies of complex multicellular and organ-level events. Slices also guarantee the presence of all of the varied cell types and cell states in the tissue, including matrix adherent cells that might be depleted during preparation of cell suspensions and organoids. Meanwhile, cell-based cultures have the benefit of higher throughput, reduced variability between replicates, and increased homogeneity, making them particularly well suited for drug screening, high-throughput analysis, and studies of cell-specific behaviors that are not dependent on architecture.

### 4.3 Models built from the bottom up in engineered systems

In some cases, additional environmental control of the culture is desirable, such as that obtainable from structured cultures in a transwell system, microdevice, or bioreactor. For example, in response to the poor correlation between results from animal vaccine studies and human clinical trials, in 2009 Byers et al. (196) developed the MIMIC™ culture system to test the efficacy of potential vaccine candidates. The system comprised a carefully proportioned co-culture of T cells, B cells and autologous DCs in 24-well plates. Cell priming with the DTaP vaccine resulted in elevated tetanus toxoid-specific class-switched B cells and IgG secretion. IgG responses in the MIMIC™ culture system were comparable to those obtained in vivo, including predicting the loss of responsiveness of aged cells to influenza vaccination (197). More recently, the two-chamber microfluidic system mentioned in section 3.3 similarly generated IgG after a recall challenge with inactivated influenza vaccines (163).

First reported in 2006 by Giese et al. (198), the Human Artificial Lymph Node (HuALN) model was one of the earliest models to replicate specific organ-level functions. This bioreactor-based model combines 3D culture in a hydrogel matrix, varied cell types, constant media perfusion, and compatibility of in-process controls for daily monitoring along with end-point analysis. Subsequent work tested the effect of vaccination with a commercial Hepatitis A viral vaccine in this system (199). A mixture of PBMCs (T cells, B cells and monocytes), mature DCs and viral inoculum was cultured for 14-30 days, resulting in activation-induced self-assembly of organoid structures and plasma cell formation, though not antibody class switching or affinity maturation. Follow-up work by Sardi et al. (200) incorporated FRC-like stromal cells in the 3D cultures, leading to the formation of a characteristic stromal network that supported T cells and DCs and increased proinflammatory cytokine secretion after antigen challenge, when compared to co-cultures of T cells and DCs alone. This bioreactor setup was also able to predict the long-term immunogenicity of monoclonal antibodies, a common challenge for immunotherapies (201).

## 5 Discussion and reflection on future opportunities

In summary, immunologists are experiencing a period of great growth in available tools and technologies, as part of the thriving field of “immunoengineering” at the convergence of immunology with the physical sciences, math, and engineering. The nascent set of in vitro, ex vivo, and computational models of the LN already have the potential to reveal immune mechanisms in the human organ that cannot be achieved any other way. However, much work remains to be done before models of immunity are able to fully predict immune function in health and disease. In this final section, we look at major gaps and opportunities for further development. Given the plethora of organs-on-chip developed in the past ten years for nearly every other organ, it may be tempting to ask why there have not been more organ-level replicas of the LN in microphysiological systems. However, a better question is to ask what groundwork has been laid for this challenging organ system. Critical advances have been made in the past 20 - 25 years. Advances in understanding of the nuances of lymphocyte phenotype and the role of the LN stroma make the biological requirements of the system clearer. There has been progress on identifying conditions for long term culture of primary human cells under stimulating conditions, for example as part of the HuALN project (198–200). Engineered cell lines provide useful simplifications in some cases, such as murine fibroblasts that were modified to express CD40L (mimicking Tfh cells) and B cell activating factor (mimicking FDCs) (161, 162). In addition, sophisticated biomaterial-based microenvironments now support largely biomimetic immune cell migration (97, 122). Hybrid systems combining LN explants with microfluidics have been developed, offering an complement to bottom-up systems (183, 184), although these are usually not held in culture for longer than a few days due to lymphocyte egress. On the computational front, physical properties (e.g. permeability) and biochemical properties (e.g. chemokine gradients) of the LN microenvironment have been simulated with models, although experimental data are still scarce. There has also been success in modeling individual cell-cell interactions, especially pair-wise interactions. And of course, robust techniques for engineered cultures and organ-on-chip systems that were developed for other organ systems provide a launchpad for modern work on the LN.

With these developments, it may finally be possible to assemble a variety of engineered models of key organ-level features of the LN. Challenges specific to LN models, versus other organs, include (i) the rich variety of cell types, cell-cell interactions, and cell-matrix interactions required to capture the response to challenges like vaccination, infection, or autoimmunity, (ii) the influx of cells and antigens from two separate vascular networks (lymphatic and blood), (iii) the motility of lymphocytes (which is different than the more stationary nature of tissue-resident cells in common organ-on-chip models such as lung, liver, brain, kidney, etc), (iv) the weeks-long culture time scales needed to generate a primary antibody response, versus the relatively short lifetime of naive primary lymphocytes in culture, and not least (v) the challenge of recruiting the right combination of immunological, engineering, and physical science expertise to tackle it. A few specific areas for ongoing development include improvements in cell sourcing and culture, better measurements of in vivo LN behavior during immune responses, and the development of new tools and technology for analysis and control of the engineered cultures. Each of these areas is addressed briefly below.

Cell sourcing for models of immune function remains a bottleneck, particularly given the diversity of cells involved. As discussed in detail in Hammel et al. (79), potential sources range from primary blood- and tissue-derived cells, to induced pluripotent stem cells, to immortalized cell lines, and each of these sources has strengths and drawbacks. A unique consideration when modeling human adaptive immune function is the potential for HLA (human leukocyte antigen) mismatch between cells that are sourced from different donors, which could cause an unintended allogeneic immune response. Mixing cells from different sexes may also potentially lead to reactivity against sex-linked antigens. HLA-matching and sex-matching have not been explored yet in most bioengineered models of immune function, as they have been easily avoided when working with inbred animals, when excluding T cells, and in short-term cultures. Meanwhile, LN stromal cells are particularly limited in terms of sourcing, as these cannot be obtained from the blood. Currently, thymus and bone marrow stromal cells are often used in place of LN stromal cells. As the LN has several stromal cell subtypes (5), and most are not yet represented in engineered cultures, it would be useful to develop methods for reproducible isolation, characterization and ex vivo/in vitro culture of specific LN stromal cell subsets, such as FDCs. Alternatives such as iPSC-derived LN stromal cell lines also would be beneficial. Finally, as the LN stroma changes during disease, efforts to characterize these changes and model them are critical (202). Because tumor-draining lymph nodes and tertiary lymphoid structures near tumors are routinely removed during surgery for some cancers (e.g. breast, melanoma), these tissues may serve potentially serve as sources of stromal cells in a tumor-draining context.

Advances in spatially resolved measurement of physical and chemical properties of the LN in its native state are needed to provide data against which to validate new models. Properties of interest include fluid flow rates, pH, oxygen, fuels such as glucose and glutamate, metabolites such as lactate, local concentrations of cytokines and growth factors, and neurotransmitters. In some cases, these may be measurable ex vivo in tissue explants (172, 181, 186, 203). However, to avoid inescapable changes that occur when the organ is severed from the lymphatic and blood vasculature, much of it will need to be measured in vivo, e.g. by noninvasive imaging (MRI, PET), moderately invasive live two-photon microscopy, or in rapidly frozen or fixed tissue (204, 205). Recent advances in spatial biology, including spatial RNAseq, high-content immunofluorescence imaging, and mass spec imaging, will undoubtedly provide valuable datasets to benchmark models against (19, 206–214). Computational models may be needed to interpret available data, for example to dissect experimental measurements of cell localization or migration in response to bound and free chemokines in the local microenvironment. In addition to characterizing the LN at rest, far more work is needed to provide spatiotemporal data on the changes that occur in the LN in response to inflammation and disease, antigen engagement, vaccination, tumor drainage, and so on.

Several advances in tool and technology development would enable better LN models. Improved techniques for real time tracking of cell-cell interactions and molecular signals would greatly increase the information compared to endpoint assays or occasional measurement of secreted proteins. In terms of environmental control, methods are needed to test the impact of intermittent rather than constant fluid flow in experimental and computational models. More research incorporating stromal cells is needed so that cell behavior and immune events can be studied in the context of the structural support and chemical signaling that the stroma provides. Furthermore, existing models of lymphatic vessels and/or blood vessels could be integrated with the LN model, to characterize cell-endothelium/stroma crosstalk and role of the LN microenvironment in disease progression (215, 216). Once models of the LN advance sufficiently, integration of additional biology will be an exciting next step. To date, most organ-level LN models have attempted to replicate humoral responses, with a focus on IgG secretion and germinal center development. In contrast, engineered organ-level models of the development of cytotoxic CD8+ T cell responses have been far less developed, but will be critical for studies of induction of antiviral or anti-tumor immunity (217). There is also a need for a LN model that can truly predict immunogenicity after vaccination with new antigens; this is a challenge due to the low precursor frequency of antigen-specific T cells. Finally, a future research direction may include integration of models of innervation of the LN. This is a nascent area of immunological research; innervation of the spleen by the vagus nerve has been shown to have major impacts on inflammatory disease and is in fact a therapeutic target (218–221). Recently, neurons in the LN were mapped out (51, 52), and work by Ross et al. (186) demonstrated spontaneous release of neurotransmitters in the LN. Far more work is needed to develop and use models to predict CD8-mediated immunity, immunogenicity, and the role of innervation in LN function.

Finally, beyond models of the LN in isolation, there is enormous potential to connect models of the LN with models of the lymphatic and blood vasculature and other organs. A shared limitation of existing LN models (explants, organoids, bioreactors, microphysiological models) is that they do not replicate the connections to the tissues they would drain in vivo; most models are isolated by design. Beyond the loss of fluid flow, this isolation means they cannot replicate the events of antigen influx or DC or lymphocyte migration into the node. An exciting future direction is to build on recent advances in multi-organ microphysiological models in the organ-on-chip field (222–224), to add elements of this connectivity to LN models. For example, connecting a LN model with a model of the brain, lung, or gut would enable study of immune responses in neurodegenerative diseases, respiratory infection, or food allergy, respectively – just to name a few examples. Similarly, there is a great deal of interest in the role of the sentinel LN during tumor growth and metastasis; this biological system is an obvious fit for multi-organ models of immunity. Such systems have the potential to be used both for testing mechanistic hypotheses, and for testing drugs and immunotherapies. Current challenges here include those that are universal to all multi-organ microphysiological systems, including the need for a common media for different organ cultures or an intact endothelial barrier to maintain distinct media compartments. Current multi-organ systems are not yet plug-and-play, but this may change rapidly over the next five years. Specific to modeling LN interactions with other organs, lymphocyte trafficking between organs, along with selective entry and egress, will be essential to establishing biomimetic interactions. Microscale pump systems that are compatible with lymphocyte recirculation will be of particular interest for this purpose (225). In addition, most fluidic connections between organs-on-chip are made by simple tubing or channels, which do not replicate the control over rate or distribution of antigen and cell influx that is provided by the lymphatic vasculature. Achieving such control may require incorporation of lymphatic barriers in well-defined locations. As LN models continue to advance, we anticipate that integrating them into multi-organ systems will become the next scientific frontier.

## Author contributions

All authors contributed to conception and design of the review, wrote portions of the first draft, and contributed to initial figure design. TO led the revision of the majority of the manuscript and drafted final figures. All authors contributed to manuscript revision, read, and approved the submitted version.

## References

[1] Grant SM Lou M Yao L Germain RN Radtke AJ. The lymph node at a glance – how spatial organization optimizes the immune response. J Cell Sci (2020) 133(5):jcs241828. doi: 10.1242/jcs.241828 32144196 PMC7063836

[2] Van den Broeck W Derore A Simoens P. Anatomy and nomenclature of murine lymph nodes: descriptive study and nomenclatory standardization in BALB/cAnNCrl mice. J Immunol Methods (2006) 312(1):12–9. doi: 10.1016/j.jim.2006.01.022 16624319

[3] Kawashima Y Sugimura M Hwang Y-C Kudo N. The lymph system in mice. Japanese J Vet Res (1964) 12(4):69–78. doi: 10.14943/jjvr.12.4.69

[4] Bajénoff M Egen JG Koo LY Laugier JP Brau F Glaichenhaus N. Stromal cell networks regulate lymphocyte entry, migration, and territoriality in lymph nodes. Immunity (2006) 25(6):989–1001. doi: 10.1016/j.immuni.2006.10.011 17112751 PMC2692293

[5] Krishnamurty AT Turley SJ. Lymph node stromal cells: cartographers of the immune system. Nat Immunol (2020) 21(4):369–80. doi: 10.1038/s41590-020-0635-3 32205888

[6] Elmore SA Bouknight SA. Lymph node. In: Parker GA, editor. Immunopathology in toxicology and drug development: volume 2, organ systems. Cham: Springer International Publishing (2017). p. 59–79. doi: 10.1007/978-3-319-47385-7_3

[7] Bajénoff M Glaichenhaus N Germain RN. Fibroblastic reticular cells guide T lymphocyte entry into and migration within the splenic T cell zone. J Immunol (2008) 181(6):3947–54. doi: 10.4049/jimmunol.181.6.3947 PMC2596721 18768849

[8] Mempel TR Henrickson SE von Andrian UH. T-Cell priming by dendritic cells in lymph nodes occurs in three distinct phases. Nature (2004) 427(6970):154–9. doi: 10.1038/nature02238 14712275

[9] Miller MJ Wei SH Cahalan MD Parker I. Autonomous T cell trafficking examined in vivo with intravital two-photon microscopy. PNAS (2003) 100(5):2604–9. doi: 10.1073/pnas.2628040100 PMC151387 12601158

[10] Bousso P Robey E. Dynamics of CD8 + T cell priming by dendritic cells in intact lymph nodes. Nat Immunol (2003) 4(6):579–85. doi: 10.1038/ni928 12730692

[11] Miller MJ Wei SH Parker I Cahalan MD. Two-photon imaging of lymphocyte motility and antigen response in intact lymph node. Science (2002) 296(5574):1869–73. doi: 10.1126/science.1070051 12016203

[12] Stoll S Delon J Brotz TM Germain RN. Dynamic imaging of T cell-dendritic cell interactions in lymph nodes. Science (2002) 296(5574):1873–6. doi: 10.1126/science.1071065 12052961

[13] Qi H Egen JG Huang AYC Germain RN. Extrafollicular activation of lymph node b cells by antigen-bearing dendritic cells. Science (2006) 312(5780):1672–6. doi: 10.1126/science.1125703 16778060

[14] Phan TG Grigorova I Okada T Cyster JG. Subcapsular encounter and complement-dependent transport of immune complexes by lymph node b cells. Nat Immunol (2007) 8(9):992–1000. doi: 10.1038/ni1494 17660822

[15] Victora GD Schwickert TA Fooksman DR Kamphorst AO Meyer-Hermann M Dustin ML. Germinal center dynamics revealed by multiphoton microscopy using a photoactivatable fluorescent reporter. Cell (2010) 143(4):592–605. doi: 10.1016/j.cell.2010.10.032 21074050 PMC3035939

[16] Victora GD Nussenzweig MC. Germinal centers. Annu Rev Immunol (2012) 30(1):429–57. doi: 10.1146/annurev-immunol-020711-075032 22224772

[17] Schwickert TA Lindquist RL Shakhar G Livshits G Skokos D Kosco-Vilbois MH. In vivo imaging of germinal centres reveals a dynamic open structure. Nature (2007) 446(7131):83–7. doi: 10.1038/nature05573 17268470

[18] Boscardin SB Hafalla JCR Masilamani RF Kamphorst AO Zebroski HA Rai U. Antigen targeting to dendritic cells elicits long-lived T cell help for antibody responses. J Exp Med (2006) 203(3):599–606. doi: 10.1084/jem.20051639 16505139 PMC2118236

[19] Goltsev Y Samusik N Kennedy-Darling J Bhate S Hale M Vazquez G. Deep profiling of mouse splenic architecture with CODEX multiplexed imaging. Cell (2018) 174(4):968–981.e15. doi: 10.1016/j.cell.2018.07.010 30078711 PMC6086938

[20] Black S Phillips D Hickey JW Kennedy-Darling J Venkataraaman VG Samusik N. CODEX multiplexed tissue imaging with DNA-conjugated antibodies. Nat Protoc (2021) 16(8):3802–35. doi: 10.1038/s41596-021-00556-8 PMC8647621 34215862

[21] Xia C Fan J Emanuel G Hao J Zhuang X. Spatial transcriptome profiling by MERFISH reveals subcellular RNA compartmentalization and cell cycle-dependent gene expression. Proc Natl Acad Sci (2019) 116(39):19490–9. doi: 10.1073/pnas.1912459116 PMC6765259 31501331

[22] Tripodo C Zanardi F Iannelli F Mazzara S Vegliante M Morello G. A spatially resolved dark- versus light-zone microenvironment signature subdivides germinal center-related aggressive b cell lymphomas. iScience (2020) 23(10):101562. doi: 10.1016/j.isci.2020.101562 33083730 PMC7522121

[23] Moysi E Del Rio Estrada PM Torres-Ruiz F Reyes-Terán G Koup RA Petrovas C. *In Situ* characterization of human lymphoid tissue immune cells by multispectral confocal imaging and quantitative image analysis; implications for HIV reservoir characterization. Front Immunol (2021) 12:683396. doi: 10.3389/fimmu.2021.683396 34177929 PMC8221112

[24] Huang HY Rivas-Caicedo A Renevey F Cannelle H Peranzoni E Scarpellino L. Identification of a new subset of lymph node stromal cells involved in regulating plasma cell homeostasis. Proc Natl Acad Sci (2018) 115(29):E6826–35. doi: 10.1073/pnas.1712628115 PMC6055158 29967180

[25] Fletcher AL Malhotra D Acton SE Lukacs-Kornek V Bellemare-Pelletier A Curry M. Reproducible isolation of lymph node stromal cells reveals site-dependent differences in fibroblastic reticular cells. Front Immun (2011) 2:35(35). doi: 10.3389/fimmu.2011.00035 PMC3342056 22566825

[26] Nakamura K Yamaji T Crocker PR Suzuki A Hashimoto Y. Lymph node macrophages, but not spleen macrophages, express high levels of unmasked sialoadhesin: implication for the adhesive properties of macrophages in vivo. Glycobiology (2002) 12(3):209–16. doi: 10.1093/glycob/12.3.209 11971865

[27] Knowlden SA Capece T Popovic M Chapman TJ Rezaee F Kim M. Regulation of T cell motility In vitro and In vivo by LPA and LPA2. PloS One (2014) 9(7):e101655. doi: 10.1371/journal.pone.0101655 25003200 PMC4086949

[28] Katakai T Kondo N Ueda Y Kinashi T. Autotaxin produced by stromal cells promotes LFA-1–independent and rho-dependent interstitial T cell motility in the lymph node paracortex. J Immunol (2014) 193(2):617–26. doi: 10.4049/jimmunol.1400565 24935929

[29] Xiong Y Brinkman CC Famulski KS Mongodin EF Lord CJ Hippen KL. A robust in vitro model for trans-lymphatic endothelial migration. Sci Rep (2017) 7(1):1633. doi: 10.1038/s41598-017-01575-w 28487567 PMC5431648

[30] Bertho N Adamski H Toujas L Debove M Davoust J Quillien V. Efficient migration of dendritic cells toward lymph node chemokines and induction of TH1 responses require maturation stimulus and apoptotic cell interaction. Blood (2005) 106(5):1734–41. doi: 10.1182/blood-2004-10-3991 15899913

[31] Freeley M O’Dowd F Paul T Kashanin D Davies A Kelleher D. L-plastin regulates polarization and migration in chemokine-stimulated human T lymphocytes. J Immunol (2012) 188(12):6357–70. doi: 10.4049/jimmunol.1103242 22581862

[32] Ingulli E Ulman DR Lucido MM Jenkins MK. *In Situ* analysis reveals physical interactions between CD11b+ dendritic cells and antigen-specific CD4 T cells after subcutaneous injection of antigen. J Immunol (2002) 169(5):2247–52. doi: 10.4049/jimmunol.169.5.2247 12193689

[33] Piccio L Vermi W Boles KS Fuchs A Strader CA Facchetti F. Adhesion of human T cells to antigen-presenting cells through SIRPβ2-CD47 interaction costimulates T-cell proliferation. Blood (2005) 105(6):2421–7. doi: 10.1182/blood-2004-07-2823 15383453

[34] Link A Vogt TK Favre S Britschgi MR Acha-Orbea H Hinz B. Fibroblastic reticular cells in lymph nodes regulate the homeostasis of naive T cells. Nat Immunol (2007) 8(11):1255–65. doi: 10.1038/ni1513 17893676

[35] Fletcher AL Acton SE Knoblich K. Lymph node fibroblastic reticular cells in health and disease. Nat Rev Immunol (2015) 15(6):350–61. doi: 10.1038/nri3846 PMC5152733 25998961

[36] Germain RN Bajénoff M Castellino F Chieppa M Egen JG Huang AYC. Making friends in out-of-the-way places: how cells of the immune system get together and how they conduct their business as revealed by intravital imaging. Immunol Rev (2008) 221(1):163–81. doi: 10.1111/j.1600-065X.2008.00591.x 18275481

[37] Koning JJ Mebius RE. Stromal cells and immune cells involved in formation of lymph nodes and their niches. Curr Opin Immunol (2020) 64:20–5. doi: 10.1016/j.coi.2020.03.003 32325389

[38] Bellomo A Gentek R Bajénoff M Baratin M. Lymph node macrophages: scavengers, immune sentinels and trophic effectors. Cell Immunol (2018) 330:168–74. doi: 10.1016/j.cellimm.2018.01.010 29397903

[39] Kedl RM Tamburini BA. Antigen archiving by lymph node stroma: a novel function for the lymphatic endothelium. Eur J Immunol (2015) 45(10):2721–9. doi: 10.1002/eji.201545739 PMC4694630 26278423

[40] Gray EE Cyster JG. Lymph node macrophages. J Innate Immun (2012) 4(5–6):424–36. doi: 10.1159/000337007 PMC3574571 22488251

[41] Gerner MY Torabi-Parizi P Germain RN. Strategically localized dendritic cells promote rapid T cell responses to lymph-borne particulate antigens. Immunity (2015) 42(1):172–85. doi: 10.1016/j.immuni.2014.12.024 25607462

[42] Cyster JG. B cell follicles and antigen encounters of the third kind. Nat Immunol (2010) 11(11):989–96. doi: 10.1038/ni.1946 20959804

[43] Pape KA Catron DM Itano AA Jenkins MK. The humoral immune response is initiated in lymph nodes by b cells that acquire soluble antigen directly in the follicles. Immunity (2007) 26(4):491–502. doi: 10.1016/j.immuni.2007.02.011 17379546

[44] Luther SA Vogt TK Siegert S. Guiding blind T cells and dendritic cells: a closer look at fibroblastic reticular cells found within lymph node T zones. Immunol Letters (2011) 138(1):9–11. doi: 10.1016/j.imlet.2011.02.006 21333683

[45] Gretz JE Norbury CC Anderson AO Proudfoot AEI Shaw S. Lymph-borne chemokines and other low molecular weight molecules reach high endothelial venules *via* specialized conduits while a functional barrier limits access to the lymphocyte microenvironments in lymph node cortex. J Exp Med (2000) 192(10):1425–40. doi: 10.1084/jem.192.10.1425 PMC2193184 11085745

[46] Yang CY Vogt TK Favre S Scarpellino L Huang HY Tacchini-Cottier F. Trapping of naive lymphocytes triggers rapid growth and remodeling of the fibroblast network in reactive murine lymph nodes. PNAS (2014) 111(1):E109–18. doi: 10.1073/pnas.1312585111 PMC3890876 24367096

[47] Schwab SR Cyster JG. Finding a way out: lymphocyte egress from lymphoid organs. Nat Immunol (2007) 8(12):1295–301. doi: 10.1038/ni1545 18026082

[48] Grigorova IL Panteleev M Cyster JG. Lymph node cortical sinus organization and relationship to lymphocyte egress dynamics and antigen exposure. PNAS (2010) 107(47):20447–52. doi: 10.1073/pnas.1009968107 PMC2996652 21059923

[49] de Castro Pinho J Förster R. Lymph-derived neutrophils primarily locate to the subcapsular and medullary sinuses in resting and inflamed lymph nodes. Cells (2021) 10(6):1486. doi: 10.3390/cells10061486 34204825 PMC8231499

[50] Mondor I Jorquera A Sene C Adriouch S Adams RH Zhou B. Clonal proliferation and stochastic pruning orchestrate lymph node vasculature remodeling. Immunity (2016) 45(4):877–88. doi: 10.1016/j.immuni.2016.09.017 27760341

[51] Huang S Ziegler CGK Austin J Mannoun N Vukovic M Ordovas-Montanes J. Lymph nodes are innervated by a unique population of sensory neurons with immunomodulatory potential. Cell (2021) 184(2):441–459.e25. doi: 10.1016/j.cell.2020.11.028 33333021 PMC9612289

[52] Cleypool CGJ Mackaaij C Lotgerink Bruinenberg D Schurink B Bleys RLAW. Sympathetic nerve distribution in human lymph nodes. J Anatomy (2021) 239(2):282–9. doi: 10.1111/joa.13422 PMC8273593 33677834

[53] Gentek R Bajénoff M. Lymph node stroma dynamics and approaches for their visualization. Trends Immunol (2017) 38(4):236–47. doi: 10.1016/j.it.2017.01.005 28214099

[54] Cinti I Denton AE. Lymphoid stromal cells–more than just a highway to humoral immunity. Oxford Open Immunol (2021) 2(1):iqab011. doi: 10.1093/oxfimm/iqab011 PMC9914513 36845565

[55] Reif K Ekland EH Ohl L Nakano H Lipp M Förster R. Balanced responsiveness to chemoattractants from adjacent zones determines b-cell position. Nature (2002) 416(6876):94–9. doi: 10.1038/416094a 11882900

[56] Li X Zhao J Kasinath V Uehara M Jiang L Banouni N. Lymph node fibroblastic reticular cells deposit fibrosis-associated collagen following organ transplantation. J Clin Invest (2020) 130(8):4182–94. doi: 10.1172/JCI136618 PMC7410068 32597832

[57] Okada T Ngo VN Ekland EH Förster R Lipp M Littman DR. Chemokine requirements for b cell entry to lymph nodes and peyer’s patches. J Exp Med (2002) 196(1):65–75. doi: 10.1084/jem.20020201 12093871 PMC2194009

[58] Cyster JG Ansel KM Reif K Ekland EH Hyman PL Tang HL. Follicular stromal cells and lymphocyte homing to follicles. Immunol Rev (2000) 176:181–93. doi: 10.1034/j.1600-065X.2000.00618.x 11043777

[59] Imal Y Yamakawa M. Morphology, function and pathology of follicular dendritic cells. Pathol Int (1996) 46(11):807–33. doi: 10.1111/j.1440-1827.1996.tb03555.x 8970191

[60] Baptista AP Roozendaal R Reijmers RM Koning JJ Unger WW Greuter M. Lymph node stromal cells constrain immunity *via* MHC class II self-antigen presentation. eLife (2014) 3:e04433. Unanue ER, editor. doi: 10.7554/eLife.04433 25407678 PMC4270074

[61] Sun Z Burdick J. Senescence of fibroblastic reticular cells in draining lymph nodes: immunoregulation following transplantation. J Clin Invest (2020) 130(8):3965–7. doi: 10.1172/JCI139153 PMC7410040 32597831

[62] Bairati A Amante L de Petris S Pernis B. Studies on the ultrastructure of the lymph nodes. Z für Zellforschung (1964) 63(5):644–72. doi: 10.1007/BF00339912 14254757

[63] Clark SL. The reticulum of lymph nodes in mice studied with the electron microscope. Am J Anat (1962) 110:217–57. doi: 10.1002/aja.1001100303 13879732

[64] Julius MH Masuda T Herzenberg LA. Demonstration that antigen-binding cells are precursors of antibody-producing cells after purification with a fluorescence-activated cell sorter. Proc Natl Acad Sci USA (1972) 69(7):1934–8. doi: 10.1073/pnas.69.7.1934 PMC426835 4114858

[65] Crissman HA Steinkamp JA. Rapid, simultaneous measurement of DNA, protein, and cell volume in single cells from large mammalian cell populations. J Cell Biol (1973) 59(3):766–71. doi: 10.1083/jcb.59.3.766 PMC2109119 4128323

[66] Köhler G Milstein C. Continuous cultures of fused cells secreting antibody of predefined specificity. Nature (1975) 256(5517):495–7. doi: 10.1038/256495a0 1172191

[67] Jaenisch R. Germ line integration and mendelian transmission of the exogenous moloney leukemia virus. Proc Natl Acad Sci (1976) 73(4):1260–4. doi: 10.1073/pnas.73.4.1260 PMC430242 1063407

[68] Ericsson AC Crim MJ Franklin CL. A brief history of animal modeling. Mo Med (2013) 110(3):201–5. PMC3979591 23829102

[69] Saiki RK Bugawan TL Horn GT Mullis KB Erlich HA. Analysis of enzymatically amplified beta-globin and HLA-DQ alpha DNA with allele-specific oligonucleotide probes. Nature (1986) 324(6093):163–6. doi: 10.1038/324163a0 3785382

[70] Carson RT Vignali DAA. Simultaneous quantitation of 15 cytokines using a multiplexed flow cytometric assay. J Immunol Methods (1999) 227(1):41–52. doi: 10.1016/S0022-1759(99)00069-1 10485253

[71] Mempel TR Henrickson SE von Andrian UH. T-Cell priming by dendritic cells in lymph nodes occurs in three distinct phases. Nature (2004) 427(6970):154–9. doi: 10.1038/nature02238 14712275

[72] Couzin-Frankel J. Cancer immunotherapy. Science (2013) 342(6165):1432–3. doi: 10.1126/science.342.6165.1432 24357284

[73] Haslbauer JD Zinner C Stalder AK Schneeberger J Menter T Bassetti S. Vascular damage, thromboinflammation, plasmablast activation, T-cell dysregulation and pathological histiocytic response in pulmonary draining lymph nodes of COVID-19. Front Immunol (2021) 12:763098. doi: 10.3389/fimmu.2021.763098 34966385 PMC8710573

[74] Cocco G Delli Pizzi A Fabiani S Cocco N Boccatonda A Frisone A. Lymphadenopathy after the anti-COVID-19 vaccine: multiparametric ultrasound findings. Biol (Basel) (2021) 10(7):652. doi: 10.3390/biology10070652 PMC8301414 34356507

[75] Wagar LE DiFazio RM Davis MM. Advanced model systems and tools for basic and translational human immunology. Genome Med (2018) 10(1):73. doi: 10.1186/s13073-018-0584-8 30266097 PMC6162943

[76] Davis MM. A prescription for human immunology. Immunity (2008) 29(6):835. doi: 10.1016/j.immuni.2008.12.003 19100694 PMC2905652

[77] Gosselin EA Eppler HB Bromberg JS Jewell CM. Designing natural and synthetic immune tissues. Nat Mater (2018) 17(6):484–98. doi: 10.1038/s41563-018-0077-6 PMC6283404 29784994

[78] Hammel JH Cook SR Belanger MC Munson JM Pompano RR. Modeling immunity In vitro: slices, chips, and engineered tissues. Annu Rev BioMed Eng (2021) 23:461–91. doi: 10.1146/annurev-bioeng-082420-124920 PMC8277680 33872520

[79] Hammel JH Zatorski JM Cook SR Pompano RR Munson JM. Engineering in vitro immune-competent tissue models for testing and evaluation of therapeutics. Adv Drug Deliv Rev (2022) 182:114111. doi: 10.1016/j.addr.2022.114111 35031388 PMC8908413

[80] Shou Y Johnson SC Quek YJ Li X Tay A. Integrative lymph node-mimicking models created with biomaterials and computational tools to study the immune system. Materials Today Bio (2022) 14:100269. doi: 10.1016/j.mtbio.2022.100269 PMC9062348 35514433

[81] Luster AD Alon R von Andrian UH. Immune cell migration in inflammation: present and future therapeutic targets. Nat Immunol (2005) 6(12):1182–90. doi: 10.1038/ni1275 16369557

[82] Worbs T Förster R. T Cell migration dynamics within lymph nodes during steady state: an overview of extracellular and intracellular factors influencing the basal intranodal T cell motility. In: Dustin M McGavern D, editors. Visualizing immunity. Berlin, Heidelberg: Springer (2009). p. 71–105. doi: 10.1007/978-3-540-93864-4_4 19521682

[83] Pietschmann P Cush JJ Lipsky PE Oppenheimer-Marks N. Identification of subsets of human T cells capable of enhanced transendothelial migration. J Immunol (1992) 149(4):1170–8. doi: 10.4049/jimmunol.149.4.1170 1380034

[84] Lidington E Nöhammer C Dominguez M Ferry B Rose ML. Inhibition of the transendothelial migration of human lymphocytes but not monocytes by phosphodiesterase inhibitors. Clin Exp Immunol (1996) 104(1):66–71. doi: 10.1046/j.1365-2249.1996.d01-660.x 8603536 PMC2200394

[85] McGettrick HM Hunter K Moss PA Buckley CD Rainger GE Nash GB. Direct observations of the kinetics of migrating T cells suggest active retention by endothelial cells with continual bidirectional migration. J Leukocyte Biol (2009) 85(1):98–107. doi: 10.1189/jlb.0508301 18948550 PMC2626767

[86] Li Jeon N Baskaran H Dertinger SKW Whitesides GM Van De Water L Toner M. Neutrophil chemotaxis in linear and complex gradients of interleukin-8 formed in a microfabricated device. Nat Biotechnol (2002) 20(8):826–30. doi: 10.1038/nbt712 12091913

[87] µ-slide chemotaxis | reproducible chemotaxis assays. ibidi (2022). Available at: https://ibidi.com/channel-slides/9–slide-chemotaxis-ibitreat.html.

[88] Luther SA Bidgol A Hargreaves DC Schmidt A Xu Y Paniyadi J. Differing activities of homeostatic chemokines CCL19, CCL21, and CXCL12 in lymphocyte and dendritic cell recruitment and lymphoid neogenesis. J Immunol (2002) 169(1):424–33. doi: 10.4049/jimmunol.169.1.424 12077273

[89] Nandagopal S Wu D Lin F. Combinatorial guidance by CCR7 ligands for T lymphocytes migration in Co-existing chemokine fields. PloS One (2011) 6(3):e18183. doi: 10.1371/journal.pone.0018183 21464944 PMC3064588

[90] Irimia D Liu SY G Tharp W Samadani A Toner M C Poznansky M. Microfluidic system for measuring neutrophil migratory responses to fast switches of chemical gradients. Lab Chip (2006) 6(2):191–8. doi: 10.1039/B511877H PMC3763904 16450027

[91] Butler KL Ambravaneswaran V Agrawal N Bilodeau M Toner M Tompkins RG. Burn injury reduces neutrophil directional migration speed in microfluidic devices. PloS One (2010) 5(7):e11921. doi: 10.1371/journal.pone.0011921 20689600 PMC2912851

[92] Adriani G Ma D Pavesi A Kamm RD Goh ELK. A 3D neurovascular microfluidic model consisting of neurons, astrocytes and cerebral endothelial cells as a blood–brain barrier. Lab Chip (2017) 17(3):448–59. doi: 10.1039/C6LC00638H 28001148

[93] Mitra B Jindal R Lee S Xu Dong D Li L Sharma N. Microdevice integrating innate and adaptive immune responses associated with antigen presentation by dendritic cells. RSC Adv (2013) 3(36):16002–10. doi: 10.1039/c3ra41308j PMC5909707 29682279

[94] Loef EJ Sheppard HM Birch NP Dunbar PR. Live-cell microscopy reveals that human T cells primarily respond chemokinetically within a CCL19 gradient that induces chemotaxis in dendritic cells. Front Immunol (2021) 12:628090/full. doi: 10.3389/fimmu.2021.628090/full 33841411 PMC8033042

[95] Purwada A Roy K Singh A. Engineering vaccines and niches for immune modulation. Acta Biomaterialia (2014) 10(4):1728–40. doi: 10.1016/j.actbio.2013.12.036 24373907

[96] Eppler HB Jewell CM. Biomaterials as tools to decode immunity. Adv Mater (2020) 32(13):e1903367. doi: 10.1002/adma.201903367 31782844 PMC7124992

[97] Stachowiak AN Irvine DJ. Inverse opal hydrogel-collagen composite scaffolds as a supportive microenvironment for immune cell migration. J BioMed Mater Res (2008) 85A(3):815–28. doi: 10.1002/jbm.a.31661 17937415

[98] Pérez del Río E Santos F Rodriguez Rodriguez X Martínez-Miguel M Roca-Pinilla R Arís A. CCL21-loaded 3D hydrogels for T cell expansion and differentiation. Biomaterials (2020) 259:120313. doi: 10.1016/j.biomaterials.2020.120313 32829146

[99] Hernandez-Gordillo V Kassis T Lampejo A Choi G Gamboa ME Gnecco JS. Fully synthetic matrices for in vitro culture of primary human intestinal enteroids and endometrial organoids. Biomaterials (2020) 254:120125. doi: 10.1016/j.biomaterials.2020.120125 32502894 PMC8005336

[100] Pompano RR Liu W Du W Ismagilov RF. Microfluidics using spatially defined arrays of droplets in one, two, and three dimensions. Ann Rev Anal Chem (2011) 4(1):59–81. doi: 10.1146/annurev.anchem.012809.102303 21370983

[101] Chang TC Tang W Koh WJH Rettie AJE Emond MJ Monnat RJ. Microwell arrays reveal cellular heterogeneity during the clonal expansion of transformed human cells. Technol (Singap World Sci) (2015) 3(4):163–71. doi: 10.1142/S2339547815200046 PMC4854201 27158641

[102] Ahrberg CD Lee JM Chung BG. Poisson statistics-mediated particle/cell counting in microwell arrays. Sci Rep (2018) 8(1):2438. doi: 10.1038/s41598-018-20913-0 29403088 PMC5799205

[103] Faley S Seale K Hughey J Schaffer DK VanCompernolle S McKinney B. Microfluidic platform for real-time signaling analysis of multiple single T cells in parallel. Lab Chip (2008) 8(10):1700–12. doi: 10.1039/b719799c PMC4160168 18813394

[104] Dura B Dougan SK Barisa M Hoehl MM Lo CT Ploegh HL. Profiling lymphocyte interactions at the single-cell level by microfluidic cell pairing. Nat Commun (2015) 6(1):5940. doi: 10.1038/ncomms6940 25585172

[105] Micropatterned slides | spatially defined cell adhesion | ibidi (2022). Available at: https://ibidi.com/83-micropatterned-slides.

[106] Microwell arrays | cell separations | liquid handling. Platypus Technologies (2022). Available at: https://www.platypustech.com/microwell-arrays.

[107] Microwell arrays | microsurfaces (2022). Available at: https://microsurfaces.com.au/microwell.html.

[108] Swartz MA Lund AW. Lymphatic and interstitial flow in the tumor microenvironment: linking mechanobiology with immunity. Nat Rev Cancer (2012) 12:210–9. doi: 10.1038/nrc3186 22362216

[109] Rosa PM Gopalakrishnan N Ibrahim H Haug M Halaas Ø. The intercell dynamics of T cells and dendritic cells in a lymph node-on-a-chip flow device. Lab Chip (2016) 16(19):3728–40. doi: 10.1039/C6LC00702C 27560793

[110] Catron DM Itano AA Pape KA Mueller DL Jenkins MK. Visualizing the first 50 hr of the primary immune response to a soluble antigen. Immunity (2004) 21(3):341–7. doi: 10.1016/j.immuni.2004.08.007 15357945

[111] Vroomans RMA Marée AFM de Boer RJ Beltman JB. Chemotactic migration of T cells towards dendritic cells promotes the detection of rare antigens. PloS Comput Biol (2012) 8(11):e1002763. doi: 10.1371/journal.pcbi.1002763 23166480 PMC3499258

[112] Bogle G Dunbar PR. Simulating T cell motility in the lymph node paracortex with a packed lattice geometry. Immunol Cell Biol (2008) 86(8):676–87. doi: 10.1038/icb.2008.60 PMC2713783 18711399

[113] Bogle G Dunbar PR. Agent-based simulation of T-cell activation and proliferation within a lymph node. Immunol Cell Biol (2010) 88:172–9. doi: 10.1038/icb.2009.78 19884904

[114] Bogle G Dunbar PR. On-lattice simulation of T cell motility, chemotaxis, and trafficking in the lymph node paracortex. PloS One (2012) 7(9):e45258. doi: 10.1371/journal.pone.0045258 23028887 PMC3447002

[115] Azarov I Peskov K Helmlinger G Kosinsky Y. Role of T cell-To-Dendritic cell chemoattraction in T cell priming initiation in the lymph node: an agent-based modeling study. Front Immunol (2019) 10:1289. doi: 10.3389/fimmu.2019.01289 31244840 PMC6579912

[116] Mirsky HP Miller MJ Linderman JJ Kirschner DE. Systems biology approaches for understanding cellular mechanisms of immunity in lymph nodes during infection. J Theor Biol (2011) 287:160–70. doi: 10.1016/j.jtbi.2011.06.037 PMC4504675 21798267

[117] Novkovic M Onder L Cheng HW Bocharov G Ludewig B. Integrative computational modeling of the lymph node stromal cell landscape. Front Immunol (2018) 9:2428. doi: 10.3389/fimmu.2018.02428 30405623 PMC6206207

[118] Knoblich K Cruz Migoni S Siew SM Jinks E Kaul B Jeffery HC. The human lymph node microenvironment unilaterally regulates T-cell activation and differentiation. PloS Biol (2018) 16(9):e2005046. doi: 10.1371/journal.pbio.2005046 30180168 PMC6122729

[119] Malhotra D Fletcher AL Astarita J Lukacs-Kornek V Tayalia P Gonzalez SF. Transcriptional profiling of stroma from inflamed and resting lymph nodes defines immunological hallmarks. Nat Immunol (2012) 13(5):499–510. doi: 10.1038/ni.2262 22466668 PMC3366863

[120] Acton SE Farrugia AJ Astarita JL Mourão-Sá D Jenkins RP Nye E. Dendritic cells control fibroblastic reticular network tension and lymph node expansion. Nature (2014) 514(7523):498–502. doi: 10.1038/nature13814 25341788 PMC4235005

[121] Katakai T Hara T Sugai M Gonda H Shimizu A. Lymph node fibroblastic reticular cells construct the stromal reticulum *via* contact with lymphocytes. J Exp Med (2004) 200(6):783–95. doi: 10.1084/jem.20040254 PMC2211971 15381731

[122] Kim J Wu B Niedzielski SM Hill MT Coleman RM Ono A. Characterizing natural hydrogel for reconstruction of three-dimensional lymphoid stromal network to model T-cell interactions. J BioMed Mater Res A (2015) 103(8):2701–10. doi: 10.1002/jbm.a.35409 PMC4486626 25649205

[123] Novkovic M Onder L Cupovic J Abe J Bomze D Cremasco V. Topological small-world organization of the fibroblastic reticular cell network determines lymph node functionality. PloS Biol (2016) 14(7):e1002515. doi: 10.1371/journal.pbio.1002515 27415420 PMC4945005

[124] Beauchemin C Dixit NM Perelson AS. Characterizing T cell movement within lymph nodes in the absence of antigen. J Immunol (2007) 178:5505–12. doi: 10.4049/jimmunol.178.9.5505 17442932

[125] Beltman JB Marée AFM Lynch JN Miller MJ de Boer RJ. Lymph node topology dictates T cell migration behavior. J Exp Med (2007) 204(4):771–80. doi: 10.1084/jem.20061278 PMC2118562 17389236

[126] Swartz MA Hubbell JA Reddy ST. Lymphatic drainage function and its immunological implications: from dendritic cell homing to vaccine design. Semin Immunol (2008) 20(2):147–56. doi: 10.1016/j.smim.2007.11.007 18201895

[127] O’Melia MJ Lund AW Thomas SN. The biophysics of lymphatic transport: engineering tools and immunological consequences. iScience (2019) 22:28–43. doi: 10.1016/j.isci.2019.11.005 31739172 PMC6864335

[128] Swartz MA Berk DA Jain RK. Transport in lymphatic capillaries. i. macroscopic measurements using residence time distribution theory. Am J Physiology-Heart Circulatory Physiol (1996) 270(1):H324–9. doi: 10.1152/ajpheart.1996.270.1.H324 8769768

[129] Dixon JB Zawieja DC M.d AAG Coté GL. Measuring microlymphatic flow using fast video microscopy. JBO (2005) 10(6):064016. doi: 10.1117/1.2135791 16409081

[130] Galanzha EI Tuchin VV Zharov VP. In vivo integrated flow image cytometry and lymph/blood vessels dynamic microscopy. JBO (2005) 10(5):054018. doi: 10.1117/1.2060567 16292978

[131] Cooper LJ Heppell JP Clough GF Ganapathisubramani B Roose T. An image-based model of fluid flow through lymph nodes. Bull Math Biol (2015) 78(1):52–71. doi: 10.1007/s11538-015-0128-y 26690921

[132] Jafarnejad M Woodruff MC Zawieja DC Carroll MC Moore JE Jr. Modelling lymph flow and fluid exchange with blood vessels in lymph nodes. Lymphat Res Biol (2015) 13(4):234–47. doi: 10.1089/lrb.2015.0028 PMC4685511 26683026

[133] Cooper LJ Zeller-Plumhoff B Clough GF Ganapathisubramani B Roose T. Using high resolution X-ray computed tomography to create an image based model of a lymph node. J Theor Biol (2018) 449:73–82. doi: 10.1016/j.jtbi.2018.04.021 29678689

[134] Tretiakova R Setukha A Savinkov R Grebennikov D Bocharov G. Mathematical modeling of lymph node drainage function by neural network. Mathematics (2021) 9(23):3093. doi: 10.3390/math9233093

[135] Oyler-Yaniv A Oyler-Yaniv J Whitlock BM Liu Z Germain RN Huse M. A tunable diffusion-consumption mechanism of cytokine propagation enables plasticity in cell-to-cell communication in the immune system. Immunity (2017) 46(4):609–20. doi: 10.1016/j.immuni.2017.03.011 PMC5442880 28389069

[136] Ross AE Pompano RR. Diffusion of cytokines in live lymph node tissue using microfluidic integrated optical imaging. Analytica Chimica Acta (2018) 1000:205–13. doi: 10.1016/j.aca.2017.11.048 29289312

[137] Pennella F Cerino G Massai D Gallo D Falvo D’Urso Labate G Schiavi A. A survey of methods for the evaluation of tissue engineering scaffold permeability. Ann BioMed Eng (2013) 41(10):2027–41. doi: 10.1007/s10439-013-0815-5 23612914

[138] Huang JH Cárdenas-Navia LI Caldwell CC Plumb TJ Radu CG Rocha PN. Requirements for T lymphocyte migration in explanted lymph nodes. J Immunol (2007) 178(12):7747–55. doi: 10.4049/jimmunol.178.12.7747 17548612

[139] Textor J Peixoto A Henrickson SE Sinn M von Andrian UH Westermann J. Defining the quantitative limits of intravital two-photon lymphocyte tracking. PNAS (2011) 108(30):12401–6. doi: 10.1073/pnas.1102288108 PMC3145739 21734152

[140] Miteva DO Rutkowski JM Dixon JB Kilarski W Shields JD Swartz MA. Transmural flow modulates cell and fluid transport functions of lymphatic endothelium. CircRes (2010) 106(5):920–U181. doi: 10.1161/CIRCRESAHA.109.207274 PMC10994404 20133901

[141] Kawai Y Kaidoh M Yokoyama Y Ohhashi T. Pivotal roles of shear stress in the microenvironmental changes that occur within sentinel lymph nodes. Cancer Sci (2012) 103(7):1245–52. doi: 10.1111/j.1349-7006.2012.02289.x PMC7659381 22463128

[142] Tomei AA Siegert S Britschgi MR Luther SA Swartz MA. Fluid flow regulates stromal cell organization and CCL21 expression in a tissue-engineered lymph node microenvironment. J Immunol (2009) 183(7):4273–83. doi: 10.4049/jimmunol.0900835 19734211

[143] Harrison DL Fang Y Huang J. T-Cell mechanobiology: force sensation, potentiation, and translation. Front Phys (2019) 7:45/full. doi: 10.3389/fphy.2019.00045/full 32601597 PMC7323161

[144] Woolf E Grigorova I Sagiv A Grabovsky V Feigelson SW Shulman Z. Lymph node chemokines promote sustained T lymphocyte motility without triggering stable integrin adhesiveness in the absence of shear forces. Nat Immunol (2007) 8(10):1076–85. doi: 10.1038/ni1499 17721537

[145] Harris AR Yuan JX Munson JM. Assessing multiparametric drug response in tissue engineered tumor microenvironment models. Methods (2018) 134–135:20–31. doi: 10.1016/j.ymeth.2017.12.010 PMC5815918 29258924

[146] Byun CK Abi-Samra K Cho YK Takayama S. Pumps for microfluidic cell culture. ELECTROPHORESIS (2014) 35(2–3):245–57. doi: 10.1002/elps.201300205 23893649

[147] Meyvantsson I Beebe DJ. Cell culture models in microfluidic systems. Annu Rev Anal Chem (Palo Alto Calif) (2008) 1:423–49. doi: 10.1146/annurev.anchem.1.031207.113042 20636085

[148] van Duinen V Trietsch SJ Joore J Vulto P Hankemeier T. Microfluidic 3D cell culture: from tools to tissue models. Curr Opin Biotechnol (2015) 35:118–26. doi: 10.1016/j.copbio.2015.05.002 26094109

[149] Henderson AR Choi H Lee E. Blood and lymphatic vasculatures on-chip platforms and their applications for organ-specific In vitro modeling. Micromachines (2020) 11(2):147. doi: 10.3390/mi11020147 32013154 PMC7074693

[150] Greenlee JD King MR. Engineered fluidic systems to understand lymphatic cancer metastasis. Biomicrofluidics (2020) 14(1):011502. doi: 10.1063/1.5133970 32002106 PMC6986954

[151] Fathi P Esch MB. Fabrication and use of a pumpless microfluidic lymphatic vessel chip. Methods Mol Biol (2022) 2373:177–99. doi: 10.1007/978-1-0716-1693-2_11 34520013

[152] Birmingham KG O’Melia MJ Bordy S Reyes Aguilar D El-Reyas B Lesinski G. Lymph node subcapsular sinus microenvironment-On-A-Chip modeling shear flow relevant to lymphatic metastasis and immune cell homing. iScience (2020) 23(11):101751. doi: 10.1016/j.isci.2020.101751 33241198 PMC7672279

[153] Bocharov G Danilov A Vassilevski Y Marchuk GI Chereshnev VA Ludewig B. Reaction-diffusion modelling of interferon distribution in secondary lymphoid organs. Math Model Nat Phenom (2011) 6(7):13–26. doi: 10.1051/mmnp/20116702

[154] Jafarnejad M Zawieja DC Brook BS Nibbs RJB Moore JE Jr. A novel computational model predicts key regulators of chemokine gradient formation in lymph nodes and site-specific roles for CCL19 and ACKR4. J Immunol Author Choice (2017) 199(7):2291. doi: 10.4049/jimmunol.1700377 PMC5602158 28807994

[155] Sagaert X Sprangers B De Wolf-Peeters C. The dynamics of the b follicle: understanding the normal counterpart of b-cell-derived malignancies. Leukemia (2007) 21(7):1378–86. doi: 10.1038/sj.leu.2404737 17495967

[156] Mesin L Ersching J Victora GD. Germinal center b cell dynamics. Immunity (2016) 45(3):471–82. doi: 10.1016/j.immuni.2016.09.001 PMC5123673 27653600

[157] Suematsu S Watanabe T. Generation of a synthetic lymphoid tissue–like organoid in mice. Nat Biotechnol (2004) 22(12):1539–45. doi: 10.1038/nbt1039 15568019

[158] Okamoto N Chihara R Shimizu C Nishimoto S Watanabe T. Artificial lymph nodes induce potent secondary immune responses in naive and immunodeficient mice. J Clin Invest (2007) 117(4):997–1007. doi: 10.1172/JCI30379 17364025 PMC1810575

[159] Kobayashi Y Watanabe T. Gel-trapped lymphorganogenic chemokines trigger artificial tertiary lymphoid organs and mount adaptive immune responses in vivo. Front Immunol (2016) 7:316. doi: 10.3389/fimmu.2016.00316 27597851 PMC4992816

[160] Purwada A Jaiswal MK Ahn H Nojima T Kitamura D Gaharwar AK. Ex vivo engineered immune organoids for controlled germinal center reactions. Biomaterials (2015) 63:24–34. doi: 10.1016/j.biomaterials.2015.06.002 26072995 PMC4490011

[161] Purwada A Shah SB Beguelin W Melnick AM Singh A. Modular immune organoids with integrin ligand specificity differentially regulate ex vivo b cell activation. ACS Biomater Sci Eng (2017) 3(2):214–25. doi: 10.1021/acsbiomaterials.6b00474 33450794

[162] Purwada A Shah SB Béguelin W August A Melnick AM Singh A. Ex vivo synthetic immune tissues with T cell signals for differentiating antigen-specific, high affinity germinal center b cells. Biomaterials (2019) 198:27–36. doi: 10.1016/j.biomaterials.2018.06.034 30041943 PMC6355359

[163] Goyal G Prabhala P Mahajan G Bausk B Gilboa T Xie L. Ectopic lymphoid follicle formation and human seasonal influenza vaccination responses recapitulated in an organ-on-a-Chip. Adv Sci (2022) 9(14):2103241. doi: 10.1002/advs.202103241 PMC9109055 35289122

[164] Martínez MR Corradin A Klein U Álvarez MJ Toffolo GM di Camillo B. Quantitative modeling of the terminal differentiation of b cells and mechanisms of lymphomagenesis. PNAS (2012) 109(7):2672–7. doi: 10.1073/pnas.1113019109 PMC3289327 22308355

[165] Thomas MJ Klein U Lygeros J Martínez MR. A probablilistic model of the germinal center reaction. Front Immunol (2019) 10:689. doi: 10.3389/fimmu.2019.00689 31001283 PMC6456718

[166] Amodio D Cotugno N Macchiarulo G Rocca S Dimopoulos Y Castrucci MR. Quantitative multiplexed imaging analysis reveals a strong association between immunogen-specific b cell responses and tonsillar germinal center immune dynamics in children after influenza vaccination. J Immunol (2018) 200(2):538–50. doi: 10.4049/jimmunol.1701312 PMC5760299 29237774

[167] Godot V Tcherakian C Gil L Cervera-Marzal I Li G Cheng L. TLR-9 agonist and CD40-targeting vaccination induces HIV-1 envelope-specific b cells with a diversified immunoglobulin repertoire in humanized mice. PloS Pathogens (2020) 16(11):e1009025. doi: 10.1371/journal.ppat.1009025 33253297 PMC7728200

[168] Schmidt A Huber JE Sercan Alp Ö Gürkov R Reichel CA Herrmann M. Complex human adenoid tissue-based ex vivo culture systems reveal anti-inflammatory drug effects on germinal center T and b cells. EBioMedicine (2020) 53:102684. doi: 10.1016/j.ebiom.2020.102684 32114393 PMC7049648

[169] Maher D Wu X Schacker T Larson M Southern P. A model system of oral HIV exposure, using human palatine tonsil, reveals extensive binding of HIV infectivity, with limited progression to primary infection. J Infect Dis (2004) 190(11):1989–97. doi: 10.1086/425423 15529264

[170] Reif T Dyckhoff G Hohenberger R Kolbe CC Gruell H Klein F. Contact-dependent inhibition of HIV-1 replication in ex vivo human tonsil cultures by polymorphonuclear neutrophils. Cell Rep Med (2021) 2(6):100317. doi: 10.1016/j.xcrm.2021.100317 34195682 PMC8233696

[171] Vidal-Rubio B Sanchez-Carril M Oliver-Morales J González-Femandez Á Gambón-Deza F. Changes in human lymphocyte subpopulations in tonsils and regional lymph nodes of human head and neck squamous carcinoma compared to control lymph nodes. BMC Immunol (2001) 2(1):2. doi: 10.1186/1471-2172-2-2 11316463 PMC31349

[172] Belanger MC Anbaei P Dunn AF Kinman AWL Pompano RR. Spatially resolved analytical chemistry in intact, living tissues. Anal Chem (2020) 92(23):15255–62. doi: 10.1021/acs.analchem.0c03625 PMC7864589 33201681

[173] Simeone K Guay-Lord R Lateef MA Péant B Kendall-Dupont J Orimoto AM. Paraffin-embedding lithography and micro-dissected tissue micro-arrays: tools for biological and pharmacological analysis of ex vivo solid tumors. Lab Chip (2019) 19(4):693–705. doi: 10.1039/C8LC00982A 30671574

[174] Glushakova S Baibakov B Margolis LB Zimmerberg J. Infection of human tonsil histocultures: a model for HIV pathogenesis. Nat Med (1995) 1(12):1320–2. doi: 10.1038/nm1295-1320 7489416

[175] Grivel JC García M Moss WJ Margolis LB. Inhibition of HIV-1 replication in human lymphoid tissues ex vivo by measles virus. J Infect Dis (2005) 192(1):71–8. doi: 10.1086/430743 15942896

[176] Lisco A Grivel JC Biancotto A Vanpouille C Origgi F Malnati MS. Viral interactions in human lymphoid tissue: human herpesvirus 7 suppresses the replication of CCR5-tropic human immunodeficiency virus type 1 *via* CD4 modulation. J Virol (2007) 81(2):708–17. doi: 10.1128/JVI.01367-06 PMC1797468 17065205

[177] Asperti-Boursin F Real E Bismuth G Trautmann A Donnadieu E. CCR7 ligands control basal T cell motility within lymph node slices in a phosphoinositide 3–kinase– independent manner. J Exp Med (2007) 204(5):1167–79. doi: 10.1084/jem.20062079 PMC2118589 17485513

[178] Salmon H Rivas-Caicedo A Asperti-Boursin F Lebugle C Bourdoncle P Donnadieu E. Ex vivo imaging of T cells in murine lymph node slices with widefield and confocal microscopes. JoVE (Journal Visualized Experiments) (2011) 53):e3054. doi: 10.3791/3054 PMC3196165 21775968

[179] Germain RN Robey EA Cahalan MD. A decade of imaging cellular motility and interaction dynamics in the immune system. Science (2012) 336(6089):1676–81. doi: 10.1126/science.1221063 PMC3405774 22745423

[180] Katakai T Habiro K Kinashi T. Dendritic cells regulate high-speed interstitial T cell migration in the lymph node *via* LFA-1/ICAM-1. J Immunol (2013) 191(3):1188–99. doi: 10.4049/jimmunol.1300739 23817428

[181] Belanger MC Ball AG Catterton MA Kinman AWL Anbaei P Groff BD. Acute lymph node slices are a functional model system to study immunity ex vivo. ACS Pharmacol Transl Sci (2021) 4(1):128–42. doi: 10.1021/acsptsci.0c00143 PMC7887751 33615167

[182] Ball AG Belanger MC Pompano RR. Detergent wash improves vaccinated lymph node handling ex vivo. J Immunol Methods (2021) 489:112943. doi: 10.1016/j.jim.2020.112943 33333059 PMC7855487

[183] Ross AE Belanger MC Woodroof JF Pompano RR. Spatially resolved microfluidic stimulation of lymphoid tissue ex vivo. Analyst (2017) 142(4):649–59. doi: 10.1039/C6AN02042A PMC7863610 27900374

[184] Shim S Belanger MC Harris AR Munson JM Pompano RR. Two-way communication between ex vivo tissues on a microfluidic chip: application to tumor–lymph node interaction. Lab Chip (2019) 19(6):1013–26. doi: 10.1039/C8LC00957K PMC6416076 30742147

[185] Ramirez A Merwitz B Lee H Vaughan E Maisel K. Multiple particle tracking (MPT) using PEGylated nanoparticles reveals heterogeneity within murine lymph nodes and between lymph nodes at different locations. Biomater Sci (2022) 10(24):6992–7003. doi: 10.1101/2022.06.02.494550 PMC10084584 36322022

[186] Lim GN Regan SL Ross AE. Subsecond spontaneous catecholamine release in mesenteric lymph node ex vivo. J Neurochem (2020) 155(4):417–29. doi: 10.1111/jnc.15115 32602936

[187] Finetti F Capitani N Manganaro N Tatangelo V Libonati F Panattoni G. Optimization of organotypic cultures of mouse spleen for staining and functional assays. Front Immunol (2020) 11:471. doi: 10.3389/fimmu.2020.00471 32265925 PMC7105700

[188] Gonzales-Viera O Woolard KD Keel MK. Lung and lymph node explants to study the interaction between host cells and canine distemper virus. Res Vet Science (2023) 154:44–51. doi: 10.1016/j.rvsc.2022.11.004 36459718

[189] Majorova D Atkins E Martineau H Vokral I Oosterhuis D Olinga P. Use of precision-cut tissue slices as a translational model to study host-pathogen interaction. Front Vet Sci (2021) 8:686088. doi: 10.3389/fvets.2021.686088 34150901 PMC8212980

[190] Schmidt A Baumjohann D. 3D tissue explant and single-cell suspension organoid culture systems for ex vivo drug testing on human tonsil-derived T follicular helper cells. In: Graca L, editor. T-Follicular helper cells: methods and protocols. New York, NY: Springer US (2022). p. 267–88. p. p. Methods in Molecular Biology). doi: 10.1007/978-1-0716-1736-6_22 34802138

[191] Astolfi M Péant B Lateef MA Rousset N Kendall-Dupont J Carmona E. Micro-dissected tumor tissues on chip: an ex vivo method for drug testing and personalized therapy. Lab Chip (2016) 16(2):312–25. doi: 10.1039/C5LC01108F 26659477

[192] Farh KKH Marson A Zhu J Kleinewietfeld M Housley WJ Beik S. Genetic and epigenetic fine mapping of causal autoimmune disease variants. Nature (2015) 518(7539):337–43. doi: 10.1038/nature13835 PMC4336207 25363779

[193] Locci M Wu JE Arumemi F Mikulski Z Dahlberg C Miller AT. Activin a programs the differentiation of human TFH cells. Nat Immunol (2016) 17(8):976–84. doi: 10.1038/ni.3494 PMC4955732 27376469

[194] Peniche AG Osorio Y Renslo AR Frantz DE Melby PC Travi BL. Development of an ex vivo lymph node explant model for identification of novel molecules active against leishmania major. Antimicrobial Agents Chemother (2014) 58(1):78–87. doi: 10.1128/AAC.00887-13 PMC3910746 24126577

[195] Wagar LE Salahudeen A Constantz CM Wendel BS Lyons MM Mallajosyula V. Modeling human adaptive immune responses with tonsil organoids. Nat Med (2021) 27(1):125–35. doi: 10.1038/s41591-020-01145-0 PMC7891554 33432170

[196] Byers AM Tapia TM Sassano ER Wittman V. In vitro antibody response to tetanus in the MIMIC™ system is a representative measure of vaccine immunogenicity. Biologicals (2009) 37(3):148–51. doi: 10.1016/j.biologicals.2009.02.018 19272794

[197] Dauner A Agrawal P Salvatico J Tapia T Dhir V Shaik SF. The in vitro MIMIC^®^ platform reflects age-associated changes in immunological responses after influenza vaccination. Vaccine (2017) 35(41):5487–94. doi: 10.1016/j.vaccine.2017.03.099 28413134

[198] Giese C Demmler CD Ammer R Hartmann S Lubitz A Miller L. A human lymph node In vitro–challenges and progress. Artif Organs (2006) 30(10):803–8. doi: 10.1111/j.1525-1594.2006.00303.x 17026580

[199] Giese C Lubitz A Demmler CD Reuschel J Bergner K Marx U. Immunological substance testing on human lymphatic micro-organoids in vitro. J Biotechnol (2010) 148(1):38–45. doi: 10.1016/j.jbiotec.2010.03.001 20416346

[200] Sardi M Lubitz A Giese C. Modeling human immunity In vitro: improving artificial lymph node physiology by stromal cells. Appl In Vitro Toxicol (2016) 2(3):143–50. doi: 10.1089/aivt.2016.0004

[201] Kraus T Lubitz A Schließer U Giese C Reuschel J Brecht R. Evaluation of a 3D human artificial lymph node as test model for the assessment of immunogenicity of protein aggregates. J Pharm Sci (2019) 108(7):2358–66. doi: 10.1016/j.xphs.2019.02.011 30797781

[202] Gonzalez Badillo F Zisi Tegou F Masina R Wright S Scully M Harwell L. Tissue-engineered stromal reticula to study lymph node fibroblastic reticular cells in type I diabetes. Cel Mol Bioeng (2020) 13(5):419–34. doi: 10.1007/s12195-020-00627-y PMC7596159 33184575

[203] Dunn AF Catterton MA Dixon DD Pompano RR. Spatially resolved measurement of dynamic glucose uptake in live ex vivo tissues. Analytica Chimica Acta (2021) 1141:47–56. doi: 10.1016/j.aca.2020.10.027 33248661 PMC7701360

[204] Wu H Estrella V Beatty M Abrahams D El-Kenawi A Russell S. T-Cells produce acidic niches in lymph nodes to suppress their own effector functions. Nat Commun (2020) 11(1):4113. doi: 10.1038/s41467-020-17756-7 32807791 PMC7431837

[205] Bullen A Friedman RS Krummel MF. Two-photon imaging of the immune system: a custom technology platform for high-speed, multicolor tissue imaging of immune responses. In: Dustin M McGavern D, editors. Visualizing immunity. Berlin: Springer Berlin Heidelberg (2009). p. 1–29. (Current Topics in Microbiology and Immunology). 10.1007/978-3-540-93864-4_1 19521679

[206] Hu KH Eichorst JP McGinnis CS Patterson DM Chow ED Kersten K. ZipSeq: barcoding for real-time mapping of single cell transcriptomes. Nat Methods (2020) 17(8):833–43. doi: 10.1038/s41592-020-0880-2 PMC7891292 32632238

[207] Stoltzfus CR Filipek J Gern BH Olin BE Leal JM Wu Y. CytoMAP: a spatial analysis toolbox reveals features of myeloid cell organization in lymphoid tissues. Cell Rep (2020) 31(3):107523. doi: 10.1016/j.celrep.2020.107523 32320656 PMC7233132

[208] Baccin C Al-Sabah J Velten L Helbling PM Grünschläger F Hernández-Malmierca P. Combined single-cell and spatial transcriptomics reveal the molecular, cellular and spatial bone marrow niche organization. Nat Cell Biol (2020) 22(1):38–48. doi: 10.1038/s41556-019-0439-6 31871321 PMC7610809

[209] Kleshchevnikov V Shmatko A Dann E Aivazidis A King HW Li T. Cell2location maps fine-grained cell types in spatial transcriptomics. Nat Biotechnol (2022) 40(5):661–71. doi: 10.1038/s41587-021-01139-4 35027729

[210] Radtke AJ Kandov E Lowekamp B Speranza E Chu CJ Gola A. IBEX: a versatile multiplex optical imaging approach for deep phenotyping and spatial analysis of cells in complex tissues. Proc Natl Acad Sci (2020) 117(52):33455–65. doi: 10.1073/pnas.2018488117 PMC7776876 33376221

[211] Lopez R Li B Keren-Shaul H Boyeau P Kedmi M Pilzer D. DestVI identifies continuums of cell types in spatial transcriptomics data. Nat Biotechnol (2022) 40(9):1360–9. doi: 10.1038/s41587-022-01272-8 PMC9756396 35449415

[212] Abe Y Sakata-Yanagimoto M Fujisawa M Miyoshi H Suehara Y Hattori K. A single-cell atlas of non-haematopoietic cells in human lymph nodes and lymphoma reveals a landscape of stromal remodelling. Nat Cell Biol (2022) 24(4):565–78. doi: 10.1038/s41556-022-00866-3 PMC9033586 35332263

[213] Zhang J Feider CL Nagi C Yu W Carter SA Suliburk J. Detection of metastatic breast and thyroid cancer in lymph nodes by desorption electrospray ionization mass spectrometry imaging. J Am Soc Mass Spectrom (2017) 28(6):1166–74. doi: 10.1007/s13361-016-1570-2 PMC5750372 28247296

[214] Abbassi-Ghadi N Veselkov K Kumar S Huang J Jones E Strittmatter N. Discrimination of lymph node metastases using desorption electrospray ionisation-mass spectrometry imaging. Chem Commun (2014) 50(28):3661–4. doi: 10.1039/C3CC48927B 24407514

[215] Henderson AR Ilan IS Lee E. A bioengineered lymphatic vessel model for studying lymphatic endothelial cell-cell junction and barrier function. Microcirculation (2021) 28(8):e12730. doi: 10.1111/micc.12730 34569678 PMC9274261

[216] Hassell BA Goyal G Lee E Sontheimer-Phelps A Levy O Chen CS. Human organ chip models recapitulate orthotopic lung cancer growth, therapeutic responses, and tumor dormancy in vitro. Cell Rep (2017) 21(2):508–16. doi: 10.1016/j.celrep.2017.09.043 29020635

[217] Li Y Frei AW Yang EY Labrada-Miravet I Sun C Rong Y. In vitro platform establishes antigen-specific CD8+ T cell cytotoxicity to encapsulated cells *via* indirect antigen recognition. Biomaterials (2020) 256:120182. doi: 10.1016/j.biomaterials.2020.120182 32599358 PMC7480933

[218] Borovikova LV Ivanova S Zhang M Yang H Botchkina GI Watkins LR. Vagus nerve stimulation attenuates the systemic inflammatory response to endotoxin. Nature (2000) 405(6785):458–62. doi: 10.1038/35013070 10839541

[219] Rosas-Ballina M Ochani M Parrish WR Ochani K Harris YT Huston JM. Splenic nerve is required for cholinergic antiinflammatory pathway control of TNF in endotoxemia. Proc Natl Acad Sci USA (2008) 105(31):11008–13. doi: 10.1073/pnas.0803237105 PMC2504833 18669662

[220] Pavlov VA Tracey KJ. The vagus nerve and the inflammatory reflex–linking immunity and metabolism. Nat Rev Endocrinol (2012) 8(12):743–54. doi: 10.1038/nrendo.2012.189 PMC4082307 23169440

[221] Kelly MJ Breathnach C Tracey KJ Donnelly SC. Manipulation of the inflammatory reflex as a therapeutic strategy. Cell Rep Med (2022) 3(7):100696. doi: 10.1016/j.xcrm.2022.100696 35858588 PMC9381415

[222] McAleer CW Long CJ Elbrecht D Sasserath T Bridges LR Rumsey JW. Multi-organ system for the evaluation of efficacy and off-target toxicity of anticancer therapeutics. Sci Trans Med (2019) 11(497):eaav1386. doi: 10.1126/scitranslmed.aav1386 31217335

[223] Sasserath T Rumsey JW McAleer CW Bridges LR Long CJ Elbrecht D. Differential monocyte actuation in a three-organ functional innate immune system-on-a-Chip. Adv Sci (Weinh) (2020) 7(13):2000323. doi: 10.1002/advs.202000323 32670763 PMC7341107

[224] Low LA Mummery C Berridge BR Austin CP Tagle DA. Organs-on-chips: into the next decade. Nat Rev Drug Discov (2021) 20(5):345–61. doi: 10.1038/s41573-020-0079-3 32913334

[225] Cook SR Musgrove HB Throckmorton AL Pompano RR. Microscale impeller pump for recirculating flow in organs-on-chip and microreactors. Lab Chip (2022) 22(3):605–20. doi: 10.1039/D1LC01081F PMC8892988 34988560

